# Minimal fatal shocks in multistable complex networks

**DOI:** 10.1038/s41598-020-68805-6

**Published:** 2020-07-16

**Authors:** Lukas Halekotte, Ulrike Feudel

**Affiliations:** Institute for Chemistry and Biology of the Marine Environment, Carl von Ossietzky University Oldenburg, Carl-von-Ossietzky-Straße 9-11, PO box 2503, 26111 Oldenburg, Germany

**Keywords:** Complex networks, Phase transitions and critical phenomena, Ecological networks, Energy grids and networks

## Abstract

Multistability is a common phenomenon which naturally occurs in complex networks. Often one of the coexisting stable states can be identified as being the desired one for a particular application. We present here a global approach to identify the minimal perturbation which will instantaneously kick the system out of the basin of attraction of its desired state and hence induce a critical or fatal transition we call *shock-tipping*. The corresponding *Minimal Fatal Shock* is a vector whose length can be used as a global stability measure and whose direction in state space allows us to draw conclusions on weaknesses of the network corresponding to critical network motifs. We demonstrate this approach in plant–pollinator networks and the power grid of Great Britain. In both system classes, tree-like substructures appear to be the most vulnerable with respect to the minimal shock perturbation.

## Introduction

Many processes in nature can be well described in terms of nonlinear dynamical systems possessing multiple stable states for constant external conditions^1,2^. As long as perturbations are small, it is sufficient to consider the linearized problem around an attracting state to evaluate its ’local’ stability. However, in real systems, the most threatening perturbations are usually not small but large, even constituting extreme events like storms, earthquakes or financial crises^3–5^. Obviously, large perturbations in multistable systems call for a ’global’ stability paradigm.

Adequate frameworks have been introduced in the ecological literature—by Holling^6,7^—as well as in the engineering literature—by Soliman and Thompson^8^ (see^9^ for a recent review)—who both argued that the local approach to stability needs to be accompanied by non-local measures capturing the characteristics of a state’s basin of attraction. Recently, this global approach has been picked up in the field of complex networks in which multistability and large disturbances occur naturally as well^10–14^. Suitable aspects of a basin of attraction are its size, shape and depth, which until now have all been used—separately^15–19^ or in combination^20–22^—to measure the stability of multistable systems.

The suitability of a certain stability criterion for a specific problem also depends on the shape or distribution of the perturbations that occur. If perturbations are best described as noise, a suitable approach is to analyze the most likely escape path from the basin of attraction^23–25^. This path is determined by the position of the saddle point on the basin boundary possessing the lowest barrier to escape^26^. A very different situation occurs if perturbations are singular, large and abrupt. The response of nonlinear systems to such perturbations has been considered in terms of linear response theory as well as transient dynamics in climate science^27^, fluid dynamics^28,29^ and energy networks^18,30,31^. In networks, the most frequently applied characteristic to measure a system’s stability against large abrupt perturbations is the relative basin size which is usually estimated by a probabilistic measure called basin stability^32–35^. The frequent use of the basin stability approach might be justified due to its easy application and interpretation but is problematic as basins of attraction can be highly complex and/or distorted^29,36–38^. Accordingly, the sole inspection of the relative basin size could underestimate the actual risk of a critical transition due to specific perturbations.

A complementary characteristic is the shortest distance between a stable state and its basin boundary^8,17^. Remarkably, the same quantity is of interest in the field of hydrodynamics, where the corresponding direction in state space depicts the energetically most efficient perturbation—or minimal seed—that induces turbulence in a laminar flow^28,39,40^. Inspired by this minimal seed approach^41^, we employ an optimization technique to identify the smallest perturbation or shock being capable of kicking a dynamical network out of its current stable state and into the basin of another state. However, our interpretation of the critical perturbation is crucially different. We assume that the critical transition—also called tipping^42^— which the shock induces leads from a desired to an undesired state in which the system function is impaired or, in the worst case, lost. In an ecological setting, the system function could be the coexistence of species, and, hence, in accordance with its effect, we name the specific perturbation the *Minimal Fatal Shock* (MiFaS). Since tipping occurs here as a consequence of one abrupt shock, we call the related critical transition *shock-tipping* or *S-tipping*. An important advantage of computing the MiFaS constitutes in providing not only the magnitude of the shock as a quantifiable global stability measure but also its direction in state space. In a network setting, the entries of this vector can be interpreted by means of the vertices of the underlying network topology. This gives us a promising tool to identify weak points constituted by certain substructures of the network and to understand their topological and dynamical origin.

We demonstrate the MiFaS in two exemplary systems—one ecological and one technical—which lay the ground for human food and electricity supply. These are mutualistic systems of plants and pollinators^43,44^, for which until now stability analyses were primarily based on local approaches^45–48^ or on perturbations of the topology^49–51^, and power grids^13,14^, for which various recent studies—using e.g. linear reponse theory^52,53^, stochastic escape^54–56^ or the basin stability approach^32,33^—focus on unravelling the impact of network structure on network stability. Despite being dynamically crucially different, the two systems share two major similarities. Firstly, the dynamics of boths systems can well be captured within a network setting whose operating modes depend on the interplay between two different types of nodes and, secondly and more essentially, their operating modes naturally compete with alternative states which represent certain failures of the systems, namely the extinction of species and power outages. In both systems, we are able to identify and classify weak topological structures which make these systems particularly vulnerable with respect to perturbations.

## Results

### Minimal fatal shock

**Figure 1:**
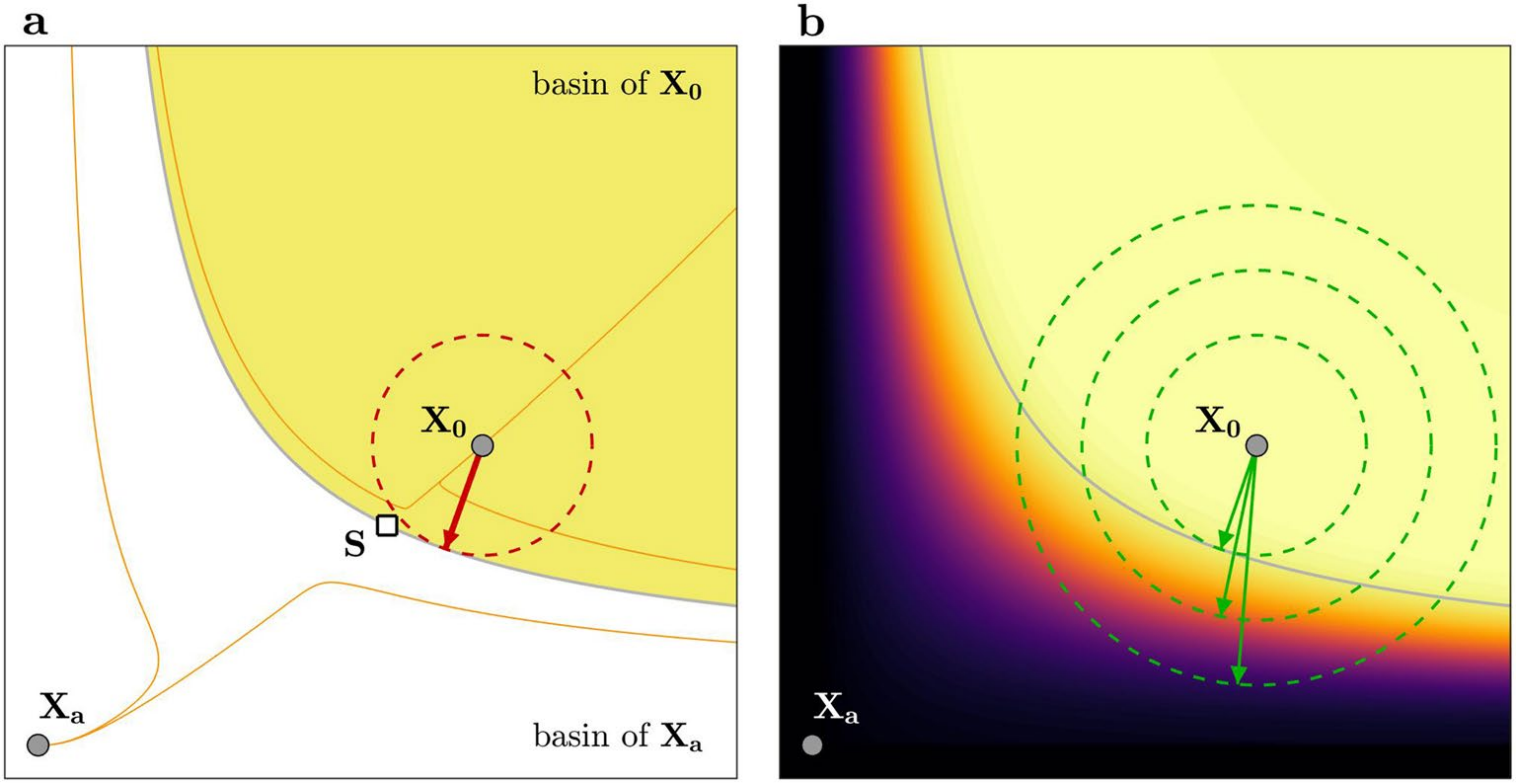
Representation of the Minimal Fatal Shock and the related search algorithm. (**a**) The MiFaS (red arrow) is the smallest perturbation to the desired state $\mathbf {X_0}$ which puts the system outside the basin of $\mathbf {X_0}$ and into the basin of an alternative attractor $\mathbf {X_a}$. (**b**) The search algorithm starts with a relatively large perturbation magnitude. The related subspace of allowed initial conditions is given by the largest circle and the direction of maximum amplification is displayed by the green arrow. As the magnitude of allowed perturbations is reduced, the direction of maximum amplification converges towards the MiFaS. Color coding marks the objective function (distance to the desired state after a short integration time) with dark colors displaying large values and bright colors small values. This figure was generated using MATLAB version R2020a (https://www.mathworks.com/).

The first step in identifying the MiFaS for a given system is to define a desired state $\mathbf {X_0}$. We then assume that, prior to perturbations, the system resides on $\mathbf {X_0}$ and that a shock—applied at $t=0$—kicks the system’s state instantaneously to $\mathbf {X}(0)$. A shock—now defined as $\mathbf {x}(0) = \mathbf {X}(0)-\mathbf {X_0}$—is said to be fatal if $\mathbf {X}(0)$ is located outside the basin of $\mathbf {X_0}$ and non-fatal if $\mathbf {X}(0)$ is located within the basin of $\mathbf {X_0}$. Accordingly, the MiFaS is a vector which displays the shortest distance between the desired state and its basin boundary and the corresponding direction in state space (Fig. 1a).

The second essential step, is defining a norm for the perturbation size. It is important to note that the use of a certain norm is not only a technical but also an interpretative decision. Throughout this work, we use the Euclidean distance to the desired state $\mathbf {X_0}$ to quantify the magnitude *d* of a perturbation

1 $$\begin{aligned} d \; = \, ||\mathbf {x}(0) || \, = \, ||\mathbf {X}(0) - \mathbf {X_0}||. \end{aligned}$$

To determine the MiFaS, we develop a search algorithm which is based on the minimal seed approach^41^ and which can be divided into two stages, the global random initialization (stage I) and the local non-random optimization (stage II).

In stage I, we randomly draw initial conditions from a shrinking subspace in state space to find a fatal shock with a preferably small magnitude *d* (see “Methods” and Supplementary Fig. S1). Stage II starts with the smallest fatal shock received from stage I (Supplementary Fig. S1). From this point on, we take two seemingly opposing steps. First, we adapt the direction of $\mathbf {x}(0)$ in order to move $\mathbf {X}(0)$ away from the basin of $\mathbf {X_0}$ while keeping *d* fixed. Second, we move $\mathbf {X}(0)$ towards the basin by reducing *d* by a step size $\Delta d$. By repeating these two steps iteratively, we attain smaller and smaller fatal shocks which finally converge towards a local MiFaS (see Fig. 1b and Supplementary Fig. S1). It is important to note that the outcome of the search—and thus the achieved local MiFaS—is dependent on the initialization in stage I. Accordingly, to attain the global MiFaS, we need to run the search algorithm multiple times and select the minimum of the local MiFaS as the global one.

**Figure 2:**
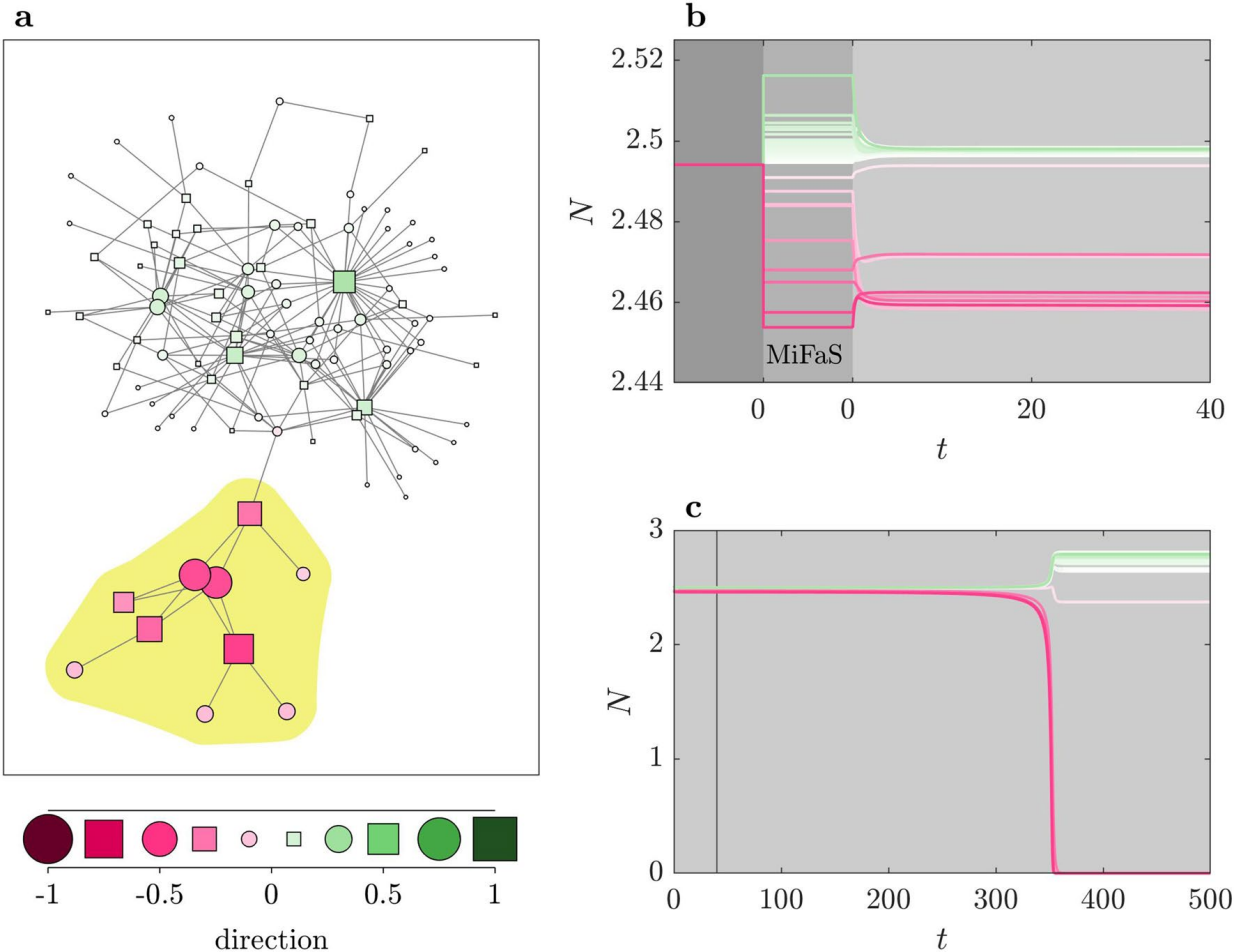
Minimal Fatal Shock for an exemplary plant–pollinator network. (**a**) Direction of the MiFaS. The perturbation vector is scaled to a length of 1. The relative contribution of each element of the vector (node in the network) to the overall perturbation is represented by the area and the color saturation of the respective squares and circles. A pink coloring denotes a loss and a green coloring a gain in species abundance at the respective node. Squares portray pollinators and circles plants. Species being lost after the perturbation are marked by the yellow shaded region. Placement of the vertices is based on the Kamada–Kawai algorithm^66^ obtained from python-igraph version 0.7.1 (https://igraph.org/). (**b**) Transient behavior following the MiFaS. Dark gray area shows the situation before the perturbation (desired state). Lighter gray area shows how the state variables are altered due to the perturbation. Light gray area depicts the transient behavior after the system has been perturbed. (**c**) Evolution over a longer time span. Vertical line displays the time interval shown in (**b**). The figure was generated using MATLAB version R2020a (https://www.mathworks.com/).

The centerpiece of the outlined algorithm is the adaptation of the direction of $\mathbf {x}(0)$ during stage II, which aims at maximizing the distance between $\mathbf {X}(0)$ and the basin boundary of $\mathbf {X_0}$. However, since this distance is not easily accessible, it is approximated by an objective function which can be maximized within a constraint optimization. For the two applications we present here, the objective function can be thought of as the amplification of the shock over a preselected time *T* (see “Methods” for specific definition). The mechanism behind this is that trajectories close to the basin boundary stay close to it for long times as they move along the stable manifold of a saddle-type state while trajectories far off the boundary approach an alternative attractor faster and thus lead to earlier and stronger amplifications.

In summary, as a result of the optimization procedure we obtain the magnitude of the smallest distance to the basin boundary which can be utilized as a quantitative measure of global stability and the direction of the perturbation in the high-dimensional phase space.

### Plant–pollinator networks

In our first example, we consider a simple model of mutualism which captures the crucial aspects of a system of plants and their corresponding pollinators^43,45^. The mutualistic system is described as a bipartite network, with one set of nodes representing a number of $N_P$ plant species and one set representing a number of $N_A$ animal species whose dynamics are given by

2 $$\begin{aligned} \frac{\mathrm {d} P_i}{\mathrm {d} t} \,&= \, \alpha P_i \, - \, \sum _{k=1}^{N_P} \beta _{ik} P_i P_k \, + \, \frac{\sum _{j=1}^{N_A} \gamma _{ij} A_j P_i}{1 + h \sum _{j=1}^{N_A} \gamma _{ij} A_j},\nonumber \\ \frac{\mathrm {d} A_j}{\mathrm {d} t} \,&= \, \alpha A_j \, - \, \sum _{l=1}^{N_A} {\tilde{\beta }}_{jl} A_j A_l \, + \, \frac{\sum _{i=1}^{N_P} {\tilde{\gamma }}_{ji} P_i A_j}{1 + h \sum _{i=1}^{N_P} {\tilde{\gamma }}_{ji} P_i}, \end{aligned}$$

where $P_i$ denotes the abundance of plant species *i* ($i=1, \ldots , N_P$) and $A_j$ the abundance of animal species *j* ($j=1, \ldots , N_A$). In Eq. (2), the parameter $\alpha$ gives the intrinsic growth rate, $\beta _{ik}$ (${\tilde{\beta }}_{jl}$) the competitive pressure of plant (animal) species *k* (*l*) on plant (animal) species *i* (*j*), $\gamma _{ij}$ (${\tilde{\gamma }}_{ji}$) the benefit plant (animal) species *i* (*j*) obtains from animal (plant) species *j* (*i*) and *h* the handling time for pollination. As a general principle, we assume the benefit a species gains from pollination to be obligatory for its own growth, an assumption which is necessary to obtain multistability in this model^57^. Therefore, we choose the net growth rate $\alpha \le 0$.

In order to keep the parametrization as simple as possible, we set $\alpha$, $\beta _{ii}$ (${\tilde{\beta }}_{jj}$) and *h* to be equal for all species. To reduce the complexity of the overall interaction pattern, we assume all-to-all coupling for the interspecific competition between species within one set, whereby $\beta _{ik}=\beta _0/(N_{P}-1)$ for $i \ne k$ (${\tilde{\beta }}_{jl}=\beta _0/(N_{A}-1)$ for $j \ne l$). By contrast, a mutualistic interaction between an animal and a plant species can either be absent, in which case $\gamma _{ij}=0$ (${\tilde{\gamma }}_{ji}=0$), or present, in which case $\gamma _{ij}=\gamma _0/\kappa _i$ (${\tilde{\gamma }}_{ji}=\gamma _0/{\tilde{\kappa }}_j$), where $\kappa _i$ (${\tilde{\kappa }}_j$) denotes the degree or the number of mutualistic partners of plant (animal) species *i* (*j*). This formulation corresponds to a full trade-off between the benefit a species attains from one partner and the number of partners this species has^45^. An important aspect of the chosen parametrization is that species solely differ on account of their position in the mutualistic network. In the following, we determine the MiFaS for realistic plant–pollinator networks from the Web of Life Database^58^ representing networks from different geographic locations across various climate zones (see Supplementary Fig. S5 and Supplementary Table S2). With $\alpha = -0.3$, $\beta _{ii}=1.0$, $\beta _0 = 1.0$, $\gamma _0 = 4.5$ and $h=0.1$, we choose the model parameters in a way that ensures that each of the studied systems possesses a state in which all species coexist. This ’desired’ state $\mathbf {X_0}$ is opposed to multiple ’undesired’ states in which one or more species are gone extinct (the MiFaS is actually fatal).

To interpret the results, it is useful to state some general considerations first. Due to the mutualism, the growth of a species depends on the abundance of its mutualistic partners. As the growth of these partners can also depend on further other partners, these further partners indirectly support the growth of the first species. We could continue building this chain of dependencies but essential is that species being close to each other within the network and especially those sharing partners benefit from each other. On the other hand, due to competition high abundances of one species directly impede the growth of all species within the same group (animals or plants). Hence, the net effect which an increase or decrease of a species’ abundance has on another species depends on the interplay between the two processes. The indirect benefits can either balance or enhance the negative effects due to competition depending on whether species are close (balance) or far apart (enhance).

At first, we compute the minimal fatal shock (MiFaS) for an exemplary network from Morant Point in Jamaica (Fig. 2a). The topology of this system is characterized by an asymmetric division into a small tree-like part and a large core, i.e. a large mostly well connected component. This topological division is mirrored in the direction of the MiFaS which is visualized by the color-coding. A small negatively perturbed part consisting of the tree-like periphery (nodes within the yellow shaded region in Fig. 2a) plus its single non-peripheral neighbor is opposed to the rest of the network which is positively perturbed. This division exemplifies how the mutualistic and competitive interactions between species shape the system’s response to perturbations. In the tree-shaped part of the network, all species are close to each other but far away from most other species. Furthermore, due to the sole connection between the two characteristic structural parts of the network, the share of partners between the two is minimal. As a result, the interdependency of species within the tree-shaped part is extremely high. Accordingly, the loss of abundance of any species in the tree-like structure—as it is the case in the MiFaS (Fig. 2)—significantly affects all other species in this tree-like periphery. On the contrary, the competitive stress due to species within the large component is high as it is not balanced by the indirect benefits. It is actually even enhanced as the increase of abundance of one species boosts the growth of its partners which again enhances the competive stress on the peripheral tree-like structure.

**Figure 3:**
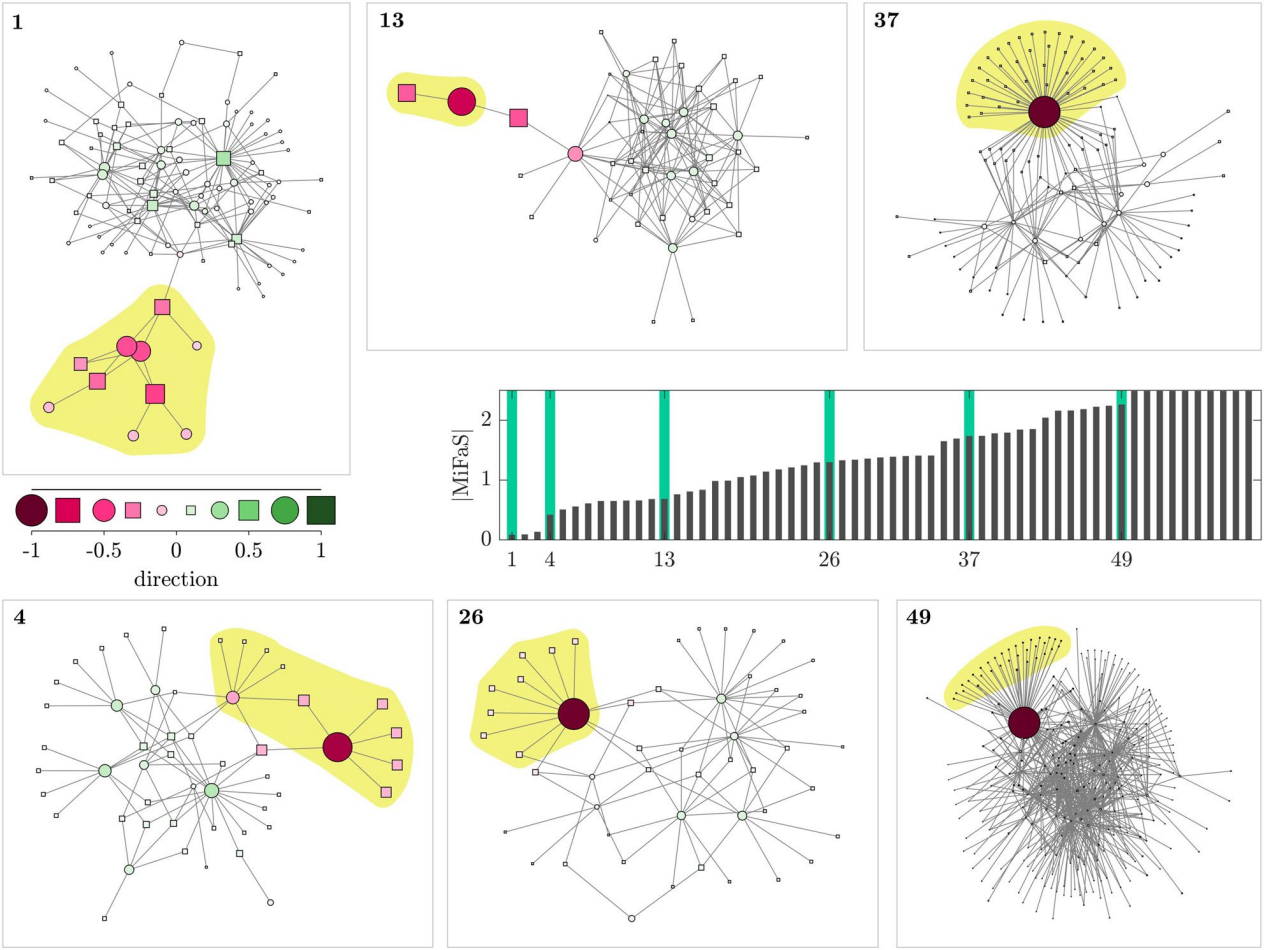
Magnitudes of 59 and direction of six MiFaS in plant–pollinator networks. The 59 networks are ordered, from low to high, and labeled according to their respective magnitude of the MiFaS. In addition, the direction of the MiFaS is shown for six exemplary networks. Perturbation vectors are scaled to a length of 1. The relative contribution of each element of the vector (node in the network) to the overall perturbation is represented by the area and the color saturation of the respective squares and circles. A pink coloring denotes a loss and a green coloring a gain in species abundance at the respective node. Squares portray pollinators and circles plants. Species being lost after the perturbation are marked by the yellow shaded region. Placement of the vertices is based on the Kamada–Kawai algorithm^66^ obtained from python-igraph version 0.7.1 (https://igraph.org/). The figure was generated using MATLAB version R2020a (https://www.mathworks.com/).

After the system has been hit by the MiFaS, all ten species within the tree-like periphery are lost in the long run (Fig. 2c and yellow shaded region in Fig. 2a). The remaining species—except for the single neighbor of the periphery—tend to higher abundances as the competitive pressure on them is relaxed. Accordingly, the new asymptotic state (Fig. 2c) again shows that the net impact of the peripheral species on most other species has been negative. Apart from the new asymptotic state, the transient leading there (Fig. 2b,c) is of interest as well. In fact, the transient behavior is typical for an initial state close to the basin boundary which is made up by the stable manifold of a saddle point. The transient at first moves towards the saddle fast (Fig. 2b), stays in its vicinity for some time as the repulsion is weak and finally settles on an attractor which, in this case, is the undesired state of partial extinction (Fig. 2c).

**Figure 4:**
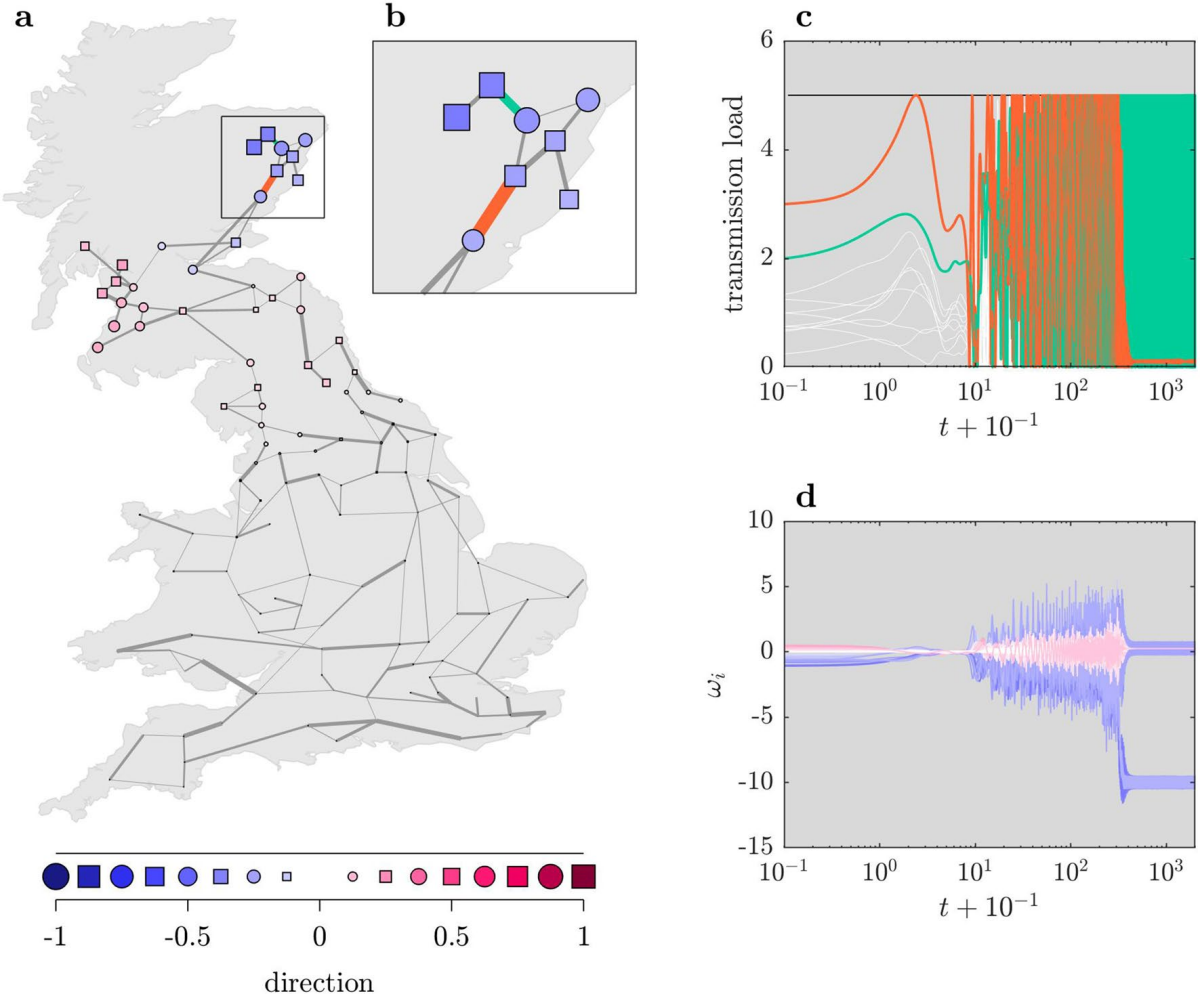
Minimal Fatal Shock in the Great Britain power grid. (**a**) Direction of the MiFaS. The perturbation vector is scaled to a length of 1. The relative contribution of each element of the vector (node in the network) to the overall perturbation is represented by the area and the color saturation of the respective squares and circles. A blue coloring denotes a deceleration and a pink coloring an acceleration at the respective node. Squares portray consumers and circles generators. Width of transmission line scales with respective initial transmission load. (**b**) Blow-up of tree-like structure in (**a**). (**c**, **d**) Transient behavior following the MiFaS. (**c**) Time series of the loads on the transmission lines included in (**b**). Colors of highlighted loads correspond to colors of transmission lines in (**b**), remaining loads are depicted in white. (**d**) Time series of the frequency deviations of all oscillators, color coding corresponds to perturbation magnitude and direction at each node. The figure was generated using MATLAB version R2020a (https://www.mathworks.com/).

Overall, we examine the MiFaS for a total of 59 plant–pollinator systems, each being based on one of the real-world network topologies. For comparison, we order the networks from sensitive to robust according to the magnitude of their respective MiFaS and depict the direction of the MiFaS for five further exemplary systems (Fig. 3).

**Figure 5:**
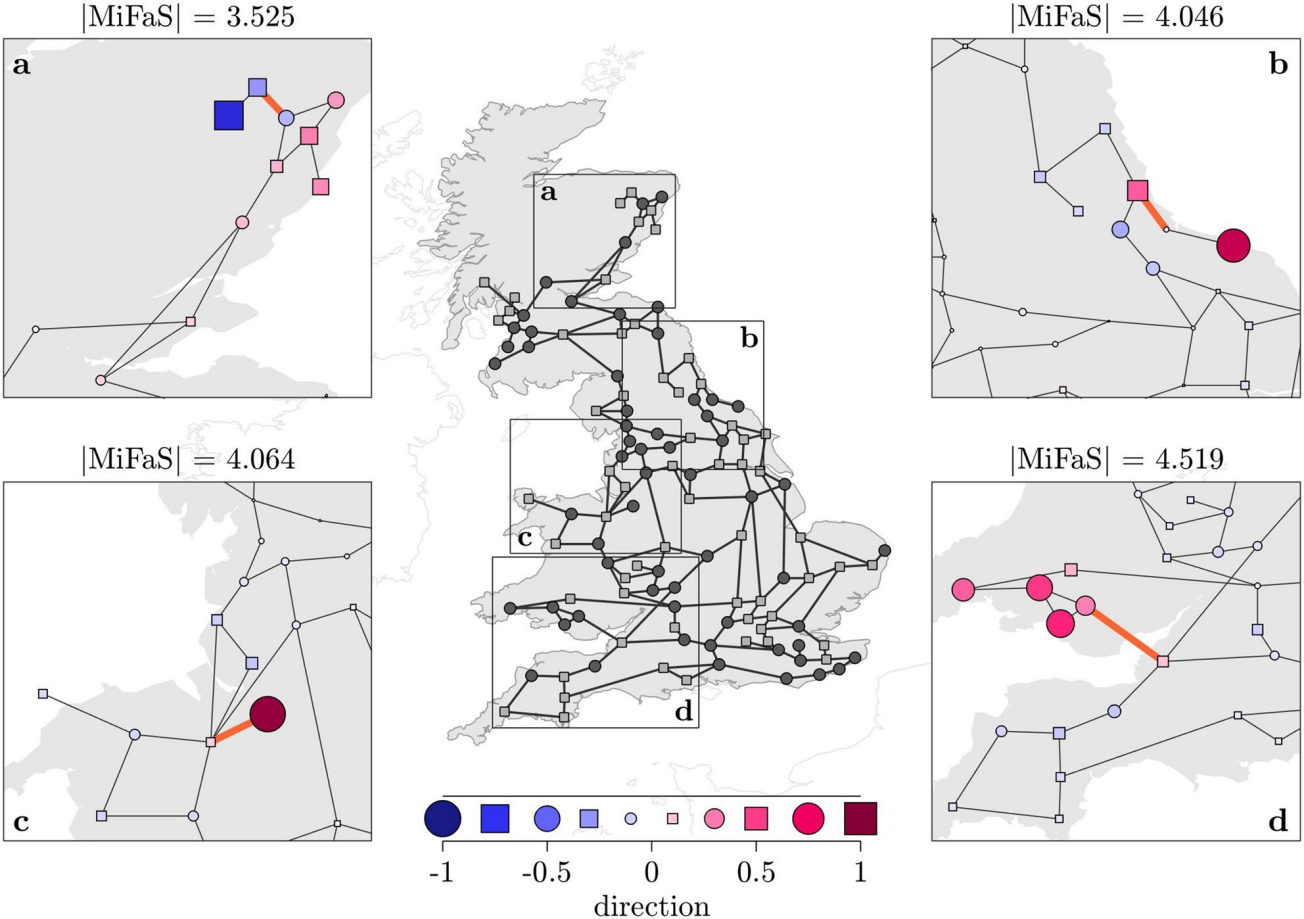
Local Minimal Fatal Shocks in the Great Britain power grid. Direction of the local MiFaS. The perturbation vectors are scaled to a length of 1. The relative contribution of each element of the vector (node in the network) to the overall perturbation is represented by the area and the color saturation of the respective squares and circles. A blue coloring denotes a deceleration and a pink coloring an acceleration at the respective node. Squares portray consumers and circles generators. (**a**–**d**) Blow-ups of the significantly perturbed area of four local MiFaS which correspond to different outcomes of the optimization process. Highlighted edges represent the trigger transmission line of the particular perturbation. The figure was generated using MATLAB version R2020a (https://www.mathworks.com/).

Some characteristics found for the MiFaS of the exemplary network (Fig. 2) prove to be generally valid. For each system, the division of the MiFaS into a small negatively perturbed part and a larger but weaker positively perturbed part displays how mutualistic interdependency and competition shape the system’s response to perturbations. In this context, the negatively perturbed part marks the weakest point of the network at whose outer edge the extinction occurs. Speaking in ecological terms, we find these weak points always being associated with specialization and the distribution of negative perturbations depends on the nature of the caused interdependency: in the exemplary system (network 1 in Fig. 3), where the specialization among all species within the tree-like structure is rather mutual, all involved species are significantly perturbed (the same for network 13 and partly for network 4, Fig. 3). However, the more asymmetric the specialization gets—meaning that many specialists are connected to a single generalist—the stronger the negative perturbation focuses on this generalist (networks 4 $\rightarrow$ 26 $\rightarrow$ 27 $\rightarrow$ 49, Fig. 3). This perturbation structure proofs to be efficient as the dependency of the generalist on each single specialist is low but its cumulated dependency on all specialized partners is high. A perturbation at the generalist therefore induces a negative feedback whose strength also depends on the number of connections the generalist has to other-non-specialized species. Accordingly, network 49 is much more robust than network 26 as the decisive generalist is highly connected to the core.

The positive contribution to the overall MiFaS marks the impact of competitive forces which depends on the global interdependency among species. In the case of a single well-connected core and a periphery which only consists of specialists being directly connected to this core, indirect positive effects between species balance competive effects as all species are close and well connected. Accordingly, we do not find any significant contribution of positive perturbations to the overall MiFaS (networks 37, 49, Fig. 3). The contrary is the case if the core is not well build, meaning that only a few connections between important hub nodes exist (networks 4, 26) or if—due to strong reciprocal specialization—a larger peripheral structure exists (networks 1, 13). In such cases, positive perturbations at rather central core-species contribute significantly to the overall MiFaS and thus to the extinction of peripheral species. In summary, a strong global interdependency among all species favors a system’s robustness whereas a strong local interdependency paired with a weak global interdependency depicts the worst case scenario.

### Great Britain power grid

As a second example we consider a coarse-grained model of a power grid which exhibits synchronization dynamics. In this framework, a power grid is described as a network of Kuramoto-like^13^ second order phase oscillators whose dynamics are given by

3 $$\begin{aligned} \frac{\mathrm {d} \phi _i}{\mathrm {d} t}&= \omega _i \nonumber \\ \frac{\mathrm {d} \omega _i}{\mathrm {d} t}&= P_i - \alpha \omega _i + \sum ^N_{j=1} K_{ji} \, \sin (\phi _j-\phi _i), \end{aligned}$$

where $\phi _i$ and $\omega _i$ denote the phase and frequency deviation of oscillator *i* from a grid’s rated frequency (which will hereinafter be referred to as phase and frequency). The parameters $\alpha$ and $P_i$ are the grid’s damping constant and the net power input/output of oscillator *i*, respectively. The capacities of the transmission lines and therefore also the topology of the grid are contained in the matrix *K*, with $K_{ji}=K_{ij}>0$ if oscillators *i* and *j* are connected and $K_{ij}=0$ otherwise.

As an example, we consider the Great Britain power grid which consists of 120 nodes and 165 transmission lines^59^. For reasons of simplification, we assume one half of the oscillators to be generators ($P_i=+P_0$) and one half to be consumers ($P_i=-P_0$) whose distribution within the grid we draw randomly (see Fig. 5). Furthermore, we choose the same maximum capacity for all transmission lines, either $K_{ij}=K_0$ or $K_{ij}=0$. In a realistic parameter setting of this model, one ’desired’ synchronized state ($\phi _i=const$ and $\omega _i=0$ for all *i*) representing stable operation competes with several ’undesired’ non-synchronized states. With $\alpha =0.1$, $P_0=1.0$ and $K_0=5.0$, we choose the model parameters accordingly. In this setting, the MiFaS represents the smallest perturbation to the synchronous state which induces a shift to one of the non-synchronous states interpreted as a power outage.

The combination of frequencies and phases is actually problematic when determining the MiFaS since they differ in units. We therefore only take into account perturbations in the frequencies $\omega$. In this context, choosing the frequencies $\omega$ instead of the phases $\phi$ seems reasonable as disturbances usually occur due to fluctuations in the power generation or consumption^60^. Such parametric disturbances would first affect the frequencies via $\mathrm {d}\omega /\mathrm {d}t$ (Eq. 3). Furthermore, considering only frequencies allows a clearer depiction of the MiFaS, since the corresponding vector contains exactly one entry per node of the power grid.

Examining one random realization of the power grid (Fig. 4a), we find that, like in the exemplary plant–pollinator network, the MiFaS is associated with a tree-like structure including the most peripheral nodes of the network (according to the resistance centrality proposed by^61^, see Supplementary Fig. S7). In fact, the same structure is highlighted by some of the eigenmodes of the graph Laplacian (see Supplementary Fig. S8). However, apart from the observation that the MiFaS is orthogonal to a neutral perturbation affecting all oscillators in the same way which is equivalent to its first eigenmode, we find no simple connection to the graph Laplacian (see Supplementary Information).

In order to understand the effectiveness of the MiFaS, it is instructive to have a closer look at how the desynchronization occurs after the system has been hit by the MiFaS (Fig. 4c,d). The desynchronization is triggered by an overload on the transmission line which connects the seven northermost oscillators to the rest of the grid (Fig. 4b). Due to the accumulation of consumers within this tree-like structure (5 consumers towards 2 generators), already in the unperturbed state, the load—$K \sin (\phi _j - \phi _i)$ for the line connecting nodes *j* and *i*—on the ’trigger transmission line’ is comparatively close to its maximum capacity *K* (see Fig. 4c). Intuitively, a strong deceleration of oscillators inside plus an acceleration of oscillators outside the tree-like structure seems to be an efficient way to induce an overload. Indeed, we find the strongest negative perturbations at the seven oscillators within (Fig. 4b) as well as positive perturbations at several oscillators outside the tree-like structure. However, in the northern part of the grid, the overall MiFaS roughly follows a broad gradient distribution with negative perturbations on both sides of the trigger transmission line and the strongest positive perturbations at rather distant nodes in the northwest of Great Britain. This distribution is efficient as the perturbations in frequencies first have to be transferred into phase deviations to induce an overload. A relatively smooth gradient ensures that the arising phase deviations are balanced slowly and thus a large transmission load can build up.

This transfer can be observed in the first stage of the transient following the MiFaS (Fig. 4c,d). In this stage, the system evolves rather smoothly towards a point where the frequency deviations of all oscillators are close to zero but where, at the same time, the transmission load on the trigger line (red line in Fig. 4) has passed its maximum capacity. The system subsequently enters a stage in which both transmission loads as well as frequencies oscillate erratically until the oscillations suddenly collapse and the system settles on an undesired attractor. It is remarkable that the final overload (green line in Fig. 4) is not located on the line which triggered the desynchronization but on a line deeper in the tree-like structure (Fig. 4c). The final overload is similar to a cutoff of two consumers from the rest of the grid, as the frequencies in the two departed components evolve more or less independently. It is however important to note that this particular undesired state represents only one of several possible outcomes. Indeed, already the slightest variation (smaller than the finite precision of the search algorithm) of the initial perturbation can lead to a different non-synchronous asymptotic state, although the trigger transmission line is always the same. Such high sensitivity is often an indicator for complexly intervowen basins of attraction, characteristic to many highly multistable systems^62^.

In order to gain more insights into how certain topological features harm a power grid’s stability against shocks, we examine some of the local MiFaS inducing power outages (Fig. 5). These local minima correspond to different outcomes of the applied optimization scheme for the same network topology and parametrization and thus represent further close but less crucial distances between the desired state and its basin boundary. As we are interested in distinct topological weak points of the grid, we take into account only those local minima which differ in the involved trigger transmission line (highlighted edges in Fig. 5).

The local MiFaS, and in particular the examination of the associated trigger transmission lines, reveal two mutually reinforcing sources for the emergence of weak points. Firstly, desynchronization events are triggered on transmission bottlenecks which result from the loose connection between a peripheral subgraph and the rest of the grid. Four out of five of the shown local MiFaS (Fig. 4 and Fig. 5a–c) are actually related to the most pronounced case of such a bottleneck which is a bridge, i.e. a single edge connecting two subnetworks. Secondly, the accumulation of oscillators of the same type within a subgraph induces a local mismatch between power generation and consumption (Fig. 4 and Fig. 5a–d). We find each of the shown local MiFaS to be related to such a local mismatch. Already in the unperturbed state, this mismatch has to be balanced by a high initial load on the connecting transmission line(s) which in turn results in a low threshold for an overload (Fig. 5d). This overload is then triggered by the MiFaS by reinforcing the generation/consumption imbalance between the two subgraphs. Accordingly, all fatal shocks involve strong frequency perturbations with a sign according to the already established power mismatch in the peripheral subgraph and frequency perturbations in the opposite direction in adjacent areas of the grid. However, as in the global MiFaS, the boundary between positive and negative perturbations is not sharp but more (Fig. 5a,c,d) or less (Fig. 5b) follows a kind of gradient.

Of particular interest is the local MiFaS shown in Fig. 5c as its underlying topological motif is quite common in the network: a node with degree 1, also termed ’dead end’^32^. Apart from the two dead ends within trees (Fig. 5a,b), the portrayed dead end is the one being most sensitive to perturbations despite or seemingly because it is connected to a rather central node of degree 6 (see also Supplementary Fig. S7). For none of the surroundings of the other dead ends, which are all adjacent to lower degree nodes, we find a local MiFaS of similar low magnitude. Accordingly, we conclude that a rather central position of the node from which the peripheral subgraph branches off might actually harm its robustness against particular perturbations.

## Discussion

One of the fundamental questions in the theory of complex networks is how structure affects stability. We address this issue from a new perspective by examining the smallest non-small perturbation which induces a critical transition towards an undesired state in a multistable system. As we treat perturbations as instantaneous state changes in phase space, we refer to them as shocks. Following an ecological interpretation of the induced *shock-tipping* or *S-tipping* event, we term the corresponding perturbation vector the Minimal Fatal Shock (MiFaS) and present an optimization algorithm to identify the MiFaS in typically high-dimensional networks. The optimization procedure provides a direction vector containing perturbation magnitudes for each node. Due to the straightforward visualization of this vector in a network setting, the MiFaS enables us to draw conclusions on structural and dynamical characteristics being responsible for the system’s high sensitivity in this particular direction. Furthermore, we show that we can combine this information with the comparison of different network topologies by using the magnitude of the MiFaS as a stability criterion. Since we are dealing with high-dimensional systems, it is difficult to prove whether the identified MiFaS is indeed the global minimal fatal shock. However, what seems to be a drawback at first sight, is in fact advantageous, namely, as we receive not only one but various dangerous perturbations which point to different topological weaknesses of a network.

We demonstrate applications of the MiFaS in two exemplary systems. The first example are pollination networks in which, to our knowledge, we are the first to tackle the issue of global stability. In the second exemplary system—power grids—the global approach to stability analyses is already much more common. Here, the MiFaS approach complements former analyses by adding the perspective of the most dangerous perturbation. Dynamically the two systems are crucially different which ultimately requires an individual formulation of the optimization problem for each system (see “Methods” and Supplementary Information). Nonetheless, the MiFaS show some prominent similarities. Most importantly, in both cases, the direction vectors of the MiFaS highlight local structures. It is this ’localization’ of the MiFaS which allows the identification of critical network motifs which account for the low stability against targeted perturbations. However, it is not only the localization but also the deduced motifs which resemble one another. We find most MiFaS being associated with intersection points—like articulation points, bridges or bridge-like edges—whose removal would disconnect or nearly disconnect a peripheral structure from the rest of the network. In fact, in both exemplary systems, the most endangered structure we find is a tree-like structure being connected to the rest of the network via a single bridge.

Nevertheless, since the nature of the edges in the two exemplary networks is different, the mechanism inducing the sensitivity against particular shocks is quite different as well. Plant–pollinator systems are interaction networks in which the edges represent the mutualism (aka pollination) which is vital for a species’ growth or reproduction. Accordingly, at the edge of the periphery, intersection points indicate high local mutualistic dependencies. These structures are vulnerable to targeted attacks as a perturbation can spread to the periphery and get stuck which ultimately induces a negative feedback loop. Responsible network motifs typically either consist of (i) several interconnected specialists or of (ii) a single generalist which is linked to many specialized species. In the latter case (ii), the generalist represents an articulation point in the network which can be translated to a ’keystone species’^50^ in an ecological sense. In case (i), the intersection is given by a bridge or a bridge-like structure which, in addition, increases the diameter of the network and thus the negative impact of competition on the peripheral species.

In power grids, the edges are supposed to balance the mismatch between generated and consumed power across the network (diffusive coupling). This process fails if the necessary load on a line exceeds its maximum capacity. To capture the effect of a MiFaS, we introduced the notion of a trigger transmission line which is the line on which the initial overload is observed. Naturally, a high initial load enhances the sensitivity of a trigger transmission line, especially if a mismatch between generated and consumed power exists within a peripheral part of the grid. Furthermore, we find that the most severe trigger transmission lines are bottlenecks connecting dead ends and other tree-like structures to the grid. This observation fits well with former studies^32,34^ in which the negative impact of these structures on network stability has been reported as well as with the recent study by Tyloo et al.^63^ which identified peripheral nodes as the ’key players’ of network synchronization (see also Supplementary Fig. S7). Due to our MiFaS approach, we observe how a perturbation in frequencies can build up phase differences which can only be balanced through a bottleneck, the trigger transmission line.

Apart from the exemplary systems presented here, the MiFaS approach is applicable to a large class of high-dimensional multistable systems, in which the global stability of a desired steady state is of crucial interest, e.g. food webs, genetic or metabolic networks or any other system which can be described by differential equations. We emphasize again that it does not only provide a quantitative measure of stability but, more importantly, directions for the most dangerous perturbations. For applications in networks this provides a tool to gain insights into the relationship between topology and stability. Specifically for interaction networks this allows to pinpoint the essential processes in the dynamics contributing to the destabilization of the desired state. Furthermore, as we have shown with the second example, the MiFaS approach works also well if not all state variables but only a subspace of perturbations is accessible—a problem relevant to many technical and natural systems. This paves the way to extent this method to single-node and multiple-node versions which enable us to compare the results with corresponding variations of other global stability techniques like basin stability^32,33,64^.

Up to now, we only discussed situations in which the proper functioning of a system is endangered by perturbations. However, the starting situation could be the exact opposite and one might be willing to actually apply the MiFaS to leave the current undesired state in favor of a desired state with minimal effort. Possible applications are the most efficient resettlement of species within an ecosystem or the combat against diseases with minimal invasive interventions. This opens another wide range of possible applications for the MiFaS.

## Methods

### Search algorithm for the MiFaS

In this section, we elaborate on the two stages of the search algorithm (outlined in “Results”) which we apply to identify a local Minimal Fatal Shock (MiFaS). A graphical representation of the two stages is given in Supplementary Fig. S1.

*Stage I: random global initialization* The first stage provides the starting point for the constraint optimization in Stage II. We start by defining a large maximum perturbation magnitude $d_{max}$. We then proceed by drawing random intial conditions $\mathbf {X}(0)$ (i.e. desired state $\mathbf {X_0}$ plus random shocks $\mathbf {x}$(0)) from a uniform distribution within the subspace centered around $\mathbf {X_0}$ and bounded by the maximum perturbation magnitude $d_{max}$^65^. By numerical integration, we check for each initial condition whether it is fatal or non-fatal. As we receive a fatal shock, we declare it the current best guess for a MiFaS. We adapt $d_{max}$ to the magnitude of the best guess and continue our random search within the now downsized subspace. After a certain number of trials ($n_{trials}$) we terminate the random search and proceed with Stage II.

*Stage II: nonrandom local search* The second stage basically consists of two things: (1) Stepwise reducing the perturbation magnitude *d* and (2) adapting the direction of the perturbation to keep it fatal. Starting from the final best guess from Stage I, we apply an optimization procedure with the perturbation magnitude *d* as a constraint (optimization on a $(n-1)$-dimensional sphere in a *n*-dimensional phase space, see Fig. 1b). The optimization aims at adapting the direction of the perturbation in order to receive system states $\mathbf {X}(0)$ as far away from the basin boundary of $\mathbf {X_0}$ as possible. However, since the distance to the boundary is not easily accessible, we need to choose an objective function to approximate it. The choice of an objective function is context dependent and an appropriate choice has to take into account the location of the desired and the undesired attractors. In the plant–pollinator networks, we use the distance after integration time *T* (see “Constraint optimization in plant–pollinator networks”) and in the power grid, we use the mean distance over the integration time *T* (see “Constraint optimization in the power grid”) which can be interpreted as the final and mean amplification of the initial perturbation. The mechanism behind this is that trajectories close to the basin boundary stay close to it for long times as they move along the stable manifold of a saddle while trajectories far away from the boundary approach an alternative attractor faster. Since in both systems, the undesired states are distant to the desired one, the latter lead to strong amplifications.

After having found the optimal perturbation $\mathbf {x}(0)$ for the initial *d*, we stepwise decrease *d* with step size $\Delta d$ and determine for each *d* the new local optimum starting from the direction of the former one. Furthermore, we check for each *d* if the corresponding optimal perturbation is still fatal. If it is, it is declared the best guess for a MiFaS. If it is not, $\Delta d$ is decreased and the procedure continues from the best guess so far. In fact, the last point is only true for the plant–pollinator system. For the power grid system, the basin landscape features fractal structures and therefore, the reduction of *d* is only terminated if the received optimum is non-fatal $n_{in}$ times consecutively. The whole procedure including the reduction of $\Delta d$ is then repeated until the requested precision of the perturbation magnitude is reached. The default parameter settings of the search algorithm are outlined in Supplementary Table S1. The dependence of computation time on network size and stability for the plant–pollinator networks is displayed in Supplementary Fig. S6 and further discussed in the Supplementary Information.

### Constraint optimization in plant–pollinator networks

In the following, we present the Lagrange multiplier approach for the plant–pollinator network. The depicted formulation generally complies with the description by Kerswell et al.^41^.

In a plant–pollinator system, the stable stationary state $\mathbf {X_0}=(P^*_1, \ldots P^*_{N_P},A^*_1 \ldots A^*_{N_A})$ with $P^*_i > 0 \forall i \in N_P$ and $A^*_j > 0 \forall j \in N_A$ is defined as the desired state, whose stability is examined. Since we use the Euclidean distance as a norm for the perturbation size (Eq. 1), it appears obvious to use it as a norm for the final distance as well. Accordingly, the objective function is defined as

4 $$\begin{aligned} o \, = \, ||\mathbf {x}(T) || \, = \, ||\mathbf {X}(T) - \mathbf {X_0}||, \end{aligned}$$

where $\mathbf {X}=(P_1, \ldots P_{N_P},A_1 \ldots A_{N_A})$ holds the state variables according to Eq. (2). As the optimization is constraint to perturbations $\mathbf {x}(0)$ with the same magnitude *d*, the objective function can be interpreted as a measure of the amplification of the initial perturbation over the integration interval *T*.

Since we are mainly interested in the distance between the system’s actual state and its desired state, it is instructive to reformulate the equations of motion to present the temporal evolution of the state variables in relation to the desired state ($\mathbf {x} := \mathbf {X}-\mathbf {X_0}$)

5 $$\begin{aligned} \frac{\mathrm {d} \mathbf {x}}{\mathrm {d} t} = \mathbf {F}(\mathbf {x};\,\mathbf {X_0}), \end{aligned}$$

which corresponds to a dynamical description of the shock evolution.

In this case, the problem of maximizing $||\mathbf {x}(T)||$ (objective functional), while simultaneously satisfying the dynamical constraint (second term) and the initial distance constraint (third term), can be formulated by the Lagrangian

6 $$\begin{aligned} {{\mathscr {L}}}(\mathbf {x},\,\mathbf {\nu },\,\lambda ;\,\mathbf {X_0},\,d,\,T) \;\; := \;\; ||\mathbf {x}(T)||^2 \;\, + \;\, \int _0^T \mathbf {\nu } \cdot \left( \frac{\mathrm {d} \mathbf {x}}{\mathrm {d}t}-\mathbf {F} \right) \mathrm {d}t \;\, + \;\, \lambda (||\mathbf {x}(0)||^2-d^2). \end{aligned}$$

where $\mathbf {\nu }(t)$ is a vector of Lagrange multipliers with the same dimension as $\mathbf {x}(t)$ and $\lambda$ is a scalar Lagrange multiplier. In order to attain maxima of the Lagrangian, first variations of ${{\mathscr {L}}}$ with respect to $\mathbf {\nu }$, $\lambda$, $\mathbf {x}(T)$, $\mathbf {x}(0)$ and $\mathbf {x}$ have to equal zero. Variations with respect to $\mathbf {\nu }$ and $\lambda$ thereby lead to the dynamical constraint (Eq. 5) and the initial distance constraint $||\mathbf {x}(0)||=d$. First variations of ${{\mathscr {L}}}$ with respect to the final and initial state vanish for

7 $$\begin{aligned} \frac{\delta {{\mathscr {L}}}}{\delta \mathbf {x}(T)} \;\;&= \;\; 2 \mathbf {x}(T) - \mathbf {\nu }(T) \;\; := \;\; 0 \end{aligned}$$

8 $$\begin{aligned} \frac{\delta {{\mathscr {L}}}}{\delta \mathbf {x}(0)} \;\;&= \;\; 2 \lambda \mathbf {x}(0) - \mathbf {\nu }(0) \;\; := \;\; 0. \end{aligned}$$

Moreover, the partial first variation of Eq. (6) with respect to *x* is given by

9 $$\begin{aligned} \frac{\delta {{\mathscr {L}}}}{\delta \mathbf {x}} \;\;\; = \;\; - \int _0^T\left[ \frac{\mathrm {d} \mathbf {\nu }}{\mathrm {d} t} + \mathbf {\nu } \cdot \frac{\partial \mathbf {F}}{\partial \mathbf {x}} \right] \mathrm {d}t, \end{aligned}$$

which is assured to equal zero for all variations of $\mathbf {x}(t)$ if

10 $$\begin{aligned} \frac{\mathrm {d} \mathbf {\nu }}{\mathrm {d} t} = - \mathbf {\nu } \cdot \frac{\partial \mathbf {F}}{\partial \mathbf {x}} \end{aligned}$$

is valid in the time interval $t \in [0,\,T]$. This expression, which is called the *adjoint* dynamical equation, describes the dynamics of the Lagrange multipliers $\mathbf {\nu }(t)$. As $\partial \mathbf {F} / \partial \mathbf {x}$ contains the partial derivatives of the actual dynamics (Eq. 5), it depends on $\mathbf {x}(t)$. For the derivation of the complete optimization problem, including the derivation of the adjoint dynamical equations, see the Supplementary Information.

In applying the set of Eqs. (5), (7), (8) and (10), and the initial distance constraint, the Lagrangian can now be maximized. For a defined value of *d*, the solution of all five equations thereby supplies a maximum, which can only be attained by an iterative method (see Supplementary Information for a detailed description of the iterative solution). First, according to equation $||\mathbf {x}(0)||=d$, an initial state is chosen and Eq. (5) is integrated forward in time. The final state $\mathbf {x}(T)$ is then applied to initialize $\mathbf {\nu }(T)$ via Eq. (7). Integrating Eq. (10) backward in time, a value for $\mathbf {\nu }(0)$ is calculated. At this point, the only condition which remains unsatisfied is Eq. (8). Therefore, in order to approach the maximum, $\mathbf {x}(0)$ is moved in the direction of $\delta {{\mathscr {L}}}/\delta \mathbf {x}(0)$ (see Supplementary Fig. S2 and notes in the Supplementary Information for a description of the step size control). Starting with the updated $\mathbf {x}(0)$, the whole procedure is repeated until the the enhancement of the current objective function is smaller than a predefined small value $o^{n+1}/o^n < 1 + \delta _{crit}$, which indicates the arrival at a maximum of ${{\mathscr {L}}}$. We note that we can not prove mathematically that this maximum is the global maximum of the optimization problem.

### Constraint optimization in the power grid

The formulation of the optimization problem for the power grid model is similar to the one in the plant–pollinator network. However, some necessary adaptations are discussed in the following.

We only consider perturbations in the frequencies $\omega$ and thus the initial distance constraint is restricted to one half of the state variables. An extra benefit is that we do not need to reformulate the model equations (see Eq. 5) to be centered around the desired state, as the desired state of complete synchronization already features $\omega _i = 0 \forall i$. Another adaptation concerns the objective function. As for the initial perturbations, we neglect phases $\phi$. However, since the system dynamics are oscillatory, we cannot rely on the Euclidean distance after a specific integration time as a corresponding objective function would be subject to strong fluctuations. Instead we use the mean Euclidean distance between the trajectory following the perturbation and the synchronized state over the integration time *T*. With these adaptations the optimization problem for the power grid can be formulated as

11 $$\begin{aligned} {{\mathscr {L}}}(\mathbf {x},\,\mathbf {\nu },\,\lambda ;\,\mathbf {X_0},\,d,\,T) \;\; := \;\; \frac{1}{T} \int _0^{T} ||\mathbf {x_f}(t)||^2 \, \mathrm {d}t \;\, + \;\, \int _0^T \mathbf {\nu } \cdot \left( \frac{\mathrm {d} \mathbf {x}}{\mathrm {d}t}-\mathbf {F} \right) \mathrm {d}t \;\, + \;\, \lambda (||\mathbf {x_f}(0)||^2-d^2) \; , \end{aligned}$$

where $\mathbf {x}=(\phi _1,\, \ldots ,\, \phi _N,\, \omega _1,\, \ldots ,\, \omega _N)$, $\mathbf {x_f}=(0,\, \ldots ,\, 0,\, \omega _1,\, \ldots ,\, \omega _N)$ and $\mathbf {F}$ holds the system’s dynamics according to Eq. (3). The derivation of the complete optimization problem can be found in the Supplementary Information.

### The choice of integration times

One of the most delicate choices in the constraint optimization procedure is the integration time *T*. Obviously, the use of short integration times is advantageous as it keeps the overall computation time short. But even more importantly, short integration times smooth differences between the magnifications caused by adjacent initial perturbations (landscape of the objective function, see Supplementary Fig. S3 and Supplementary Fig. S4) and thus allow the application of the optimization even in highly complex landscapes of basins of attractions. However, in order to resolve small scale structures close to the basin boundary, longer integration times are needed (see Supplementary Information). We solve this issue by increasing the integration time *T* during the search algorithm (whenever $\Delta d$ is decreased, see Supplementary Fig. S1 and Supplementary Table S1).

### Plant–pollinator data processing

The topologies of the 59 studied plant–pollinator networks have been taken from the Web of Life dataset (www.web.of-life.es, see Supplementary Table S2 for original data sources). In order to attain comparable, connected networks, we processed the original data as follows: (1) All networks are considered as being unweighted. (2) From every dataset, only the largest connected component is taken as the network topology while all other components are omitted.

## Supplementary information

Supplementary information.

## Data Availability

The datasets generated during the current study are available from the corresponding author upon request.

## Code availability

The computer code which has been used to determine the MiFaS for the plant–pollinator networks and the Great Britain power grid can be found at https://github.com/halekotte/mifas.
