# Supplementary material for: Minimal fatal shocks in multistable complex networks
Source: Sci Rep. 2020 Jul 16;10:11783. doi: 10.1038/s41598-020-68805-6 (PMC7366637; doi:10.1038/s41598-020-68805-6)
Supplement: Supplementary file 1 — Supplementary information. [file 41598_2020_68805_MOESM1_ESM.pdf]

Supplementary Information for

Minimal Fatal Shocks in Multistable Complex Networks

Lukas Halekotte and Ulrike Feudel

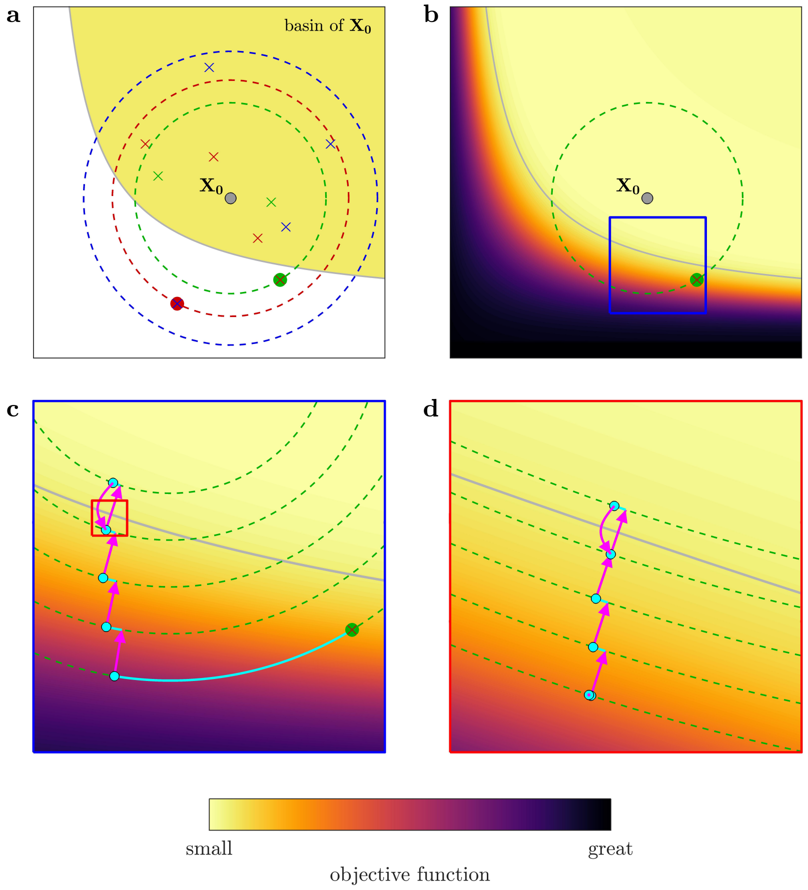

Supplementary Figure S1: **Representation of the two stages of the search algorithm for the MiFaS** (a) Within the first stage of the search algorithm, random initial conditions are drawn from a uniform distribution within the subspace centered around X_0 and bounded by a maximum perturbation magnitude d_{max} (blue circle). As soon as a fatal shock is found, it is declared the current best guess for a MiFaS and d_{max} is adapted (red circle). The search is continued in the now downsized subspace. After a number of n_{trials} trials the search is terminated. (b) The final best guess from stage I (green dot in (a) and (b)) is the starting point for the second stage II - the non-random local optimization. The blue square in (b) shows the blow up depicted in (c). (c) Within the second stage of the search algorithm, first, for a fixed d the objective function is maximized by adapting the direction of the perturbation (cyan curve). If the perturbation related to this maximum is fatal, it is declared the best guess. As the maximum is reached d is decreased by Δd (straight pink arrow). This procedure is iterated until the basin boundary (gray line) is crossed. At this point the procedure returns to the best guess (curved pink arrow). The red square in (c) shows the blow up depicted in (d). (d) Continuing from the current best guess, the procedure is repeated with a smaller Δd until the desired precision is obtained. This figure was generated using MATLAB version R2017a (`https://www.mathworks.com/`).

Supplementary Note Derivation of the Complete Optimization Problem for the Plant-Pollinator Networks

In the following, we present the complete formulation of the optimization problem for the plant-pollinator network which has already been broadly outlined in the Methods section of the main text.

The mutualistic system is described as a bipartite network, with one set of nodes representing a number of N_P plant species and one set representing a number of N_A animal species. Their dynamics are given by

$$\frac{\mathrm{d}P_i}{\mathrm{d}t} = \alpha P_i - \sum_{k=1}^{N_P} \beta_{ik} P_i P_k + \frac{\sum_{j=1}^{N_A} \gamma_{ij} A_j P_i}{1 + h \sum_{j=1}^{N_A} \gamma_{ij} A_j} ,$$
$$\frac{\mathrm{d}A_j}{\mathrm{d}t} = \alpha A_j - \sum_{l=1}^{N_A} \tilde{\beta}_{jl} A_j A_l + \frac{\sum_{i=1}^{N_P} \tilde{\gamma}_{ji} P_i A_j}{1 + h \sum_{i=1}^{N_P} \tilde{\gamma}_{ji} P_i} , \tag{S1}$$

where P_i denotes the abundance of plant species i ($i = 1, ..., N_P$) and A_j the abundance of animal species j ($j = 1, ..., N_A$). The parameter α gives the intrinsic growth rate, β_{ik} ($\tilde{\beta}_{jl}$) the competitive pressure of plant (animal) species k (l) on plant (animal) species i (j), γ_{ij} ($\tilde{\gamma}_{ji}$) the benefit plant (animal) species i (j) obtains from animal (plant) species j (i) and h the handling time for pollination. As a general principle, we assume the benefit a species gains from pollination to be obligatory for its own growth. Therefore, we choose the net growth rate α to be negative for both animal and plant species.

In this system, we assume that it is desired to maintain a state in which all species coexist and no species extinctions occur. Accordingly, we denote this stable steady state

$$\mathbf{X_0} = (P_1^*, P_2^*, ..., P_{N_P}^*, A_1^*, A_2^*, ..., A_{N_A}^*) , \tag{S2}$$

with $P_i^* > 0 \; \forall \; i \in N_P$ and $A_j^* > 0 \; \forall \; j \in N_A$ as the desired state whose stability is examined.

The general idea of the optimization is to find the perturbation to $\mathbf{X_0}$ over all perturbations with the same initial magnitude which leads to the maximum distance to $\mathbf{X_0}$ after a certain integration time. Since both norms are defined in relation to the desired state, it is instructive to describe the system dynamics in reference to $\mathbf{X_0}$ and thus we consider

$$\mathbf{x} = \mathbf{X} - \mathbf{X_0} = (p_1, p_2, ..., p_{N_P}, a_1, a_2, ..., a_{N_A}) , \tag{S3}$$

where $\mathbf{X}$ holds the original state variables described by equation (S1) and p_i and a_j are the new state variables in reference to $\mathbf{X_0}$. Since $\mathrm{d}P_i^*/\mathrm{d}t = 0$ and $\mathrm{d}A_j^*/\mathrm{d}t = 0$, the dynamics of the shifted system can be expressed in the same way as the original dynamics as

$$f_i = \frac{\mathrm{d}p_i}{\mathrm{d}t} = \alpha P_i - \sum_{k=1}^{N_P} \beta_{ik} P_i P_k + \frac{\sum_{j=1}^{N_A} \gamma_{ij} A_j P_i}{1 + h \sum_{j=1}^{N_A} \gamma_{ij} A_j}$$
$$g_j = \frac{\mathrm{d}a_j}{\mathrm{d}t} = \alpha A_j - \sum_{l=1}^{N_A} \tilde{\beta}_{jl} A_j A_l + \frac{\sum_{i=1}^{N_P} \tilde{\gamma}_{ji} P_i A_j}{1 + h \sum_{i=1}^{N_P} \tilde{\gamma}_{ji} P_i} , \tag{S4}$$

with $P_i = p_i + P_i^*$ and $A_j = a_j + A_j^*$. Accordingly, the dynamics of the complete network are captured by

$$\frac{\mathrm{d}\mathbf{x}}{\mathrm{d}t} = \mathbf{F}(\mathbf{x}; \mathbf{X_0}) = (f_1, f_2, ..., f_{N_P}, g_1, g_2, ..., g_{N_A}) . \tag{S5}$$

By stating the dynamical equations, we already formulated the dynamical constraint for the optimization problem. Another constraint concerns the set of allowed initial conditions or the subspace in phase space in

which the optimization is executed. This is complied by the initial distance constraint

$$||\mathbf{x}(0)|| = d \, , \tag{S6}$$

which equals the Euclidean distance to the desired state.

Finally, the optimization problem is completed by the objective functional o which is chosen to quantify the magnification of the initial perturbation. We choose the Euclidean distance to the desired state at time $t = T$ which equals

$$o = ||\mathbf{x}(T)|| \tag{S7}$$

Now, the problem of maximizing the objective functional (equation (S7)), while simultaneously satisfying the dynamical constraint (equation (S5)) and the initial distance constraint (equation (S6)), can be formulated by the Lagrangian

$$\mathcal{L}(\mathbf{x},\, \boldsymbol{\nu},\, \lambda;\, \mathbf{X_0},\, d,\, T) \;:=\; ||\mathbf{x}(T)||^2 \;+\; \int_0^T \boldsymbol{\nu} \cdot \left(\frac{\mathrm{d}\mathbf{x}}{\mathrm{d}t} - \mathbf{F} \right) \mathrm{d}t \;+\; \lambda(||\mathbf{x}(0)||^2 - d^2) \, . \tag{S8}$$

where $\boldsymbol{\nu}(t)$ is a vector of Lagrange multipliers with the same dimension as $\mathbf{x}(t)$ and λ is a single Lagrange multiplier. In order to attain maxima of the Lagrangian, first variations of $\mathcal{L}$ with respect to $\boldsymbol{\nu}$, λ, $\mathbf{x}(T)$, $\mathbf{x}(0)$ and $\mathbf{x}$ have to equal zero. Variations with respect to $\boldsymbol{\nu}$ and λ thereby lead to the dynamical constraint (equation (S5)) and the initial distance constraint (equation (S6)).

Variations of $\mathcal{L}$ with respect to the final and initial state vanish for

$$\frac{\delta \mathcal{L}}{\delta \mathbf{x}(T)} \;=\; 2\mathbf{x}(T) - \boldsymbol{\nu}(T) \;:=\; 0 \tag{S9}$$

$$\frac{\delta \mathcal{L}}{\delta \mathbf{x}(0)} \;=\; 2\lambda\mathbf{x}(0) - \boldsymbol{\nu}(0) \;:=\; 0 \, . \tag{S10}$$

Moreover, the partial first variation of equation (S8) with respect to x is given by

$$\frac{\delta \mathcal{L}}{\delta \mathbf{x}} \;=\; -\int_0^T \left[\frac{\mathrm{d}\boldsymbol{\nu}}{\mathrm{d}t} + \boldsymbol{\nu} \cdot \frac{\partial \mathbf{F}}{\partial \mathbf{x}} \right] \mathrm{d}t \, , \tag{S11}$$

which is assured to equal zero for all variations of $\mathbf{x}(t)$ if

$$\frac{\mathrm{d}\boldsymbol{\nu}}{\mathrm{d}t} = -\boldsymbol{\nu} \cdot \frac{\partial \mathbf{F}}{\partial \mathbf{x}} \tag{S12}$$

is valid in the time interval $t \in [0,\, T]$. This expression, which is called the *adjoint* dynamical equation, describes the dynamics of the Lagrange multipliers $\boldsymbol{\nu}(t)$. As $\partial\mathbf{F}/\partial\mathbf{x}$ contains the partial derivatives of the actual dynamics (equation (S5)), it depends on $\mathbf{x}(t)$.

In order to complete the set of equations being necessary for the solution of the optimization problem, we need to derive the explicit equations for the *adjoint* dynamics. The set of Lagrange multipliers in $\boldsymbol{\nu}$ corresponds to the state variables of the adjoint dynamical equation. In accordance with the actual state variables $\mathbf{x}$, two types of adjoint state variables (u_i and v_i) are considered and thus

$$\boldsymbol{\nu} \;=\; (u_1,\, u_2,\, ...,\, u_{N_P},\, v_1,\, v_2,\, ...,\, v_{N_A}) \, . \tag{S13}$$

In order to derive the evolution equation for the set of state variables in $\boldsymbol{\nu}$, the Jacobian matrix of the

dynamical system in equation (S4) has to be considered. The Jacobian can be outlined as

$$\frac{\partial \mathbf{F}}{\partial \mathbf{x}} = \left(\begin{array}{ccc|ccc} \frac{\partial f_1}{\partial p_1} & \cdots & \frac{\partial f_1}{\partial p_{N_P}} & \frac{\partial f_1}{\partial a_1} & \cdots & \frac{\partial f_1}{\partial a_{N_A}} \\ \vdots & \ddots & \vdots & \vdots & \ddots & \vdots \\ \frac{\partial f_{N_P}}{\partial p_1} & \cdots & \frac{\partial f_{N_P}}{\partial p_{N_P}} & \frac{\partial f_{N_P}}{\partial a_1} & \cdots & \frac{\partial f_{N_P}}{\partial a_{N_A}} \\ \frac{\partial g_1}{\partial p_1} & \cdots & \frac{\partial g_1}{\partial p_{N_P}} & \frac{\partial g_1}{\partial a_1} & \cdots & \frac{\partial g_1}{\partial a_{N_A}} \\ \vdots & \ddots & \vdots & \vdots & \ddots & \vdots \\ \frac{\partial g_{N_A}}{\partial p_1} & \cdots & \frac{\partial g_{N_A}}{\partial p_{N_P}} & \frac{\partial g_{N_A}}{\partial a_1} & \cdots & \frac{\partial g_{N_A}}{\partial a_{N_A}} \end{array}\right) . \tag{S14}$$

In accordance with equation (S12), the dynamics of one adjoint state variable are determined by the entries of one row of the Jacobian matrix. The entries of the Jacobian of the shifted plant-pollinator system (equation (S4)) can be characterized by three generic partial derivatives. The first constitutes the partial derivative of the dynamics f_i of species i with respect to itself

$$\frac{\partial f_i}{\partial p_i} = \alpha_i - \sum_{k=1}^{N_P} \beta_{ik} P_k - \beta_{ii} P_i + \frac{\sum_{j=1}^{N_A} \gamma_{ij} A_j}{1 + h \sum_{j=1}^{N_A} \gamma_{ij} A_j} . \tag{S15}$$

The second generic partial derivative applies to the partial derivatives of all species dynamics f_k within the same partition as species i

$$\frac{\partial f_k}{\partial p_i} = -\beta_{ki} P_k , \tag{S16}$$

and the third derivative captures the partial derivative of the dynamics of all species within the other partition with respect to species i

$$\frac{\partial g_j}{\partial p_i} = \frac{\tilde{\gamma}_{ji} A_j}{(1 + h \sum_{k=1}^{N_P} \tilde{\gamma}_{jk} P_k)^2} , \tag{S17}$$

where it should be noted that $\tilde{\gamma}_{ji} = 0$ if animal species j is not connected to plant species i.

Inserting equations (S15), (S16) and (S17) into equation (S12) and taking into account that any plant species i is connected to $N_P - 1$ species within the same partition and up to N_A animal species within the other partition, the adjoint dynamical equation can be derived as

$$\frac{\mathrm{d}u_i}{\mathrm{d}t} = -\left(u_i \frac{\partial f_i}{\partial p_i} + \sum_{k \neq i}^{N_P} u_k \frac{\partial f_k}{\partial p_i} + \sum_{j=1}^{N_A} v_j \frac{\partial g_j}{\partial p_i} \right) , \tag{S18}$$

which ultimately leads to the adjoint dynamical equation

$$\frac{\mathrm{d}u_i}{\mathrm{d}t} = -\alpha u_i + \sum_{k=1}^{N_P} \beta_{ik} P_k u_i + \sum_{k=1}^{N_P} \beta_{ki} P_k u_k - u_i \frac{\sum_{j=1}^{N_A} \gamma_{ij} A_j}{1 + h \sum_{j=1}^{N_A} \gamma_{ij} A_j} - \sum_{j=1}^{N_A} v_j \frac{\tilde{\gamma}_{ji} A_j}{(1 + h \sum_{k=1}^{N_P} \tilde{\gamma}_{jk} P_k)^2} \tag{S19}$$

Since the dynamics for the animal species can be written in a symmetric way to the dynamics of the plant species by interchanging a and p (as well as A and P), the adjoint dynamical equations for the Lagrange

multiplier corresponding to an animal species are

$$\frac{\mathrm{d}v_j}{\mathrm{d}t} = -\alpha v_j + \sum_{l=1}^{N_A} \tilde{\beta}_{jl} A_l v_j + \sum_{l=1}^{N_A} \tilde{\beta}_{lj} A_l v_l - v_j \frac{\sum_{i=1}^{N_P} \tilde{\gamma}_{ji} P_i}{1 + h \sum_{i=1}^{N_P} \tilde{\gamma}_{ji} P_i} - \sum_{i=1}^{N_P} u_i \frac{\gamma_{ij} P_i}{(1 + h \sum_{l=1}^{N_A} \gamma_{il} A_l)^2} \,. \tag{S20}$$

With the set of equations (S4), (S6), (S9), (S10) and (S19), (S20) the maximization problem can be solved. For a defined value of d, the solution of all equations thereby supplies a maximum, which can only be attained by an iterative method which is described in detail in the next but one Supplementary Note.

Supplementary Note Derivation of the Complete Optimization Problem for the Power Grid

The formulation of the optimization problem for the power grid model is similar to the one in the plant-pollinator network. However, some adaptations are necessary since we consider different initial perturbations and use a different objective functional.

A power grid is described as a network of Kuramoto-like [1] second order phase oscillators whose dynamics are given by

$$\begin{aligned} f_i &= \frac{\mathrm{d}\phi_i}{\mathrm{d}t} = \omega_i \\ g_i &= \frac{\mathrm{d}\omega_i}{\mathrm{d}t} = P_i - \alpha\omega_i + \sum_{j=1}^{N} K_{ji}\, \sin(\phi_j - \phi_i)\ , \end{aligned} \tag{S21}$$

where ϕ_i and ω_i denote the phase and frequency deviation of oscillator i from a grid's rated frequency (hereinafter termed phase and frequency). The parameters α and P_i are the grid's damping constant and the net power input/output of oscillator i, respectively. The capacities of the transmission lines and therefore also the topology of the grid are contained in the matrix K, with $K_{ji} = K_{ij} > 0$ if oscillators i and j are connected and $K_{ij} = 0$ otherwise.

The power grid holds two state variables for every node of the network, an oscillator's phase and frequency. Accordingly, the state of the system is hold within a vector with $2N$ entries

$$\mathbf{x} \ = \ (\phi_1,\, \phi_2,\, ...,\, \phi_N,\, \omega_1,\, \omega_2,\, ...,\, \omega_N)\ , \tag{S22}$$

where N denotes the number of nodes/oscillators within the grid. In this model, a proper system functioning is constituted by a single steady state in which all oscillators are synchronized to the grid's rated frequency and thus $\omega_i = 0\ \forall i$. We assume this state to be the desired one

$$\mathbf{x_0} \ = \ (\phi_1^*,\, \phi_2^*,\, ...,\, \phi_N^*,\, 0,\, 0,\, ...,\, 0)\ , \tag{S23}$$

with $\phi_i^* = const\ \forall i$.

The power grid model contains two different types of state variables. Combining the two within a single distance norm is problematic. We therefore only take into account the frequencies ω in the initial and final distance norm. It is important to note that this implies that we only consider perturbations in the frequencies. An extra benefit is that - in contrast to the plant-pollinator system - we do not need to shift the model equations to be centered around the desired state, as the desired state of complete synchronization already features $\omega_i = 0\ \forall\ i$. Instead, we introduce the vector

$$\mathbf{x_f} \ = \ (0,\, 0,\, ...,\, 0,\, \omega_1,\, \omega_2,\, ...,\, \omega_N) \tag{S24}$$

to formulate the initial distance constraint and the objective functional for the optimization problem.

For the initial distance constraint, we can stick to the Euclidean distance as initially only frequencies are affected by the perturbations.

$$||\mathbf{x_f}(0)|| = d \tag{S25}$$

Furthermore, we also adapt the objective functional o. Since we only consider one of two state variable types and the system dynamics are oscillatory, we cannot rely on the distance after a specific integration time T as the corresponding distance norm would be subject to strong fluctuations. Instead we use the mean Euclidean

distance between the actual and the synchronized state over the integration time T

$$o = \frac{1}{T}\int_0^T ||\mathbf{x_f}(t)||^2 \, \mathrm{d}t \, , \tag{S26}$$

With these adaptations the optimization problem for the power grid can be formulated as

$$\mathcal{L}(\mathbf{x},\, \boldsymbol{\nu},\, \lambda;\, \mathbf{X_0},\, d,\, T) \;:=\; \frac{1}{T}\int_0^T ||\mathbf{x_f}(t)||^2 \, \mathrm{d}t \;+\; \int_0^T \boldsymbol{\nu} \cdot \left(\frac{\mathrm{d}\mathbf{x}}{\mathrm{d}t} - \mathbf{F}\right) \mathrm{d}t \;+\; \lambda(||\mathbf{x_f}(0)||^2 - d^2) \, . \tag{S27}$$

In order to attain maxima, variations of $\mathcal{L}$ with respect to the Lagrange multipliers $\boldsymbol{\nu}$ and λ, the final $\mathbf{x}(T)$ and initial state $\mathbf{x_f}(0)$ and the actual state $\mathbf{x}$ have to equal zero. For variations regarding the Lagrange multipliers, the dynamical constraint and the initial distance constraint are received. Variations with respect to $\mathbf{x}(T)$ and $\mathbf{x_f}(0)$ lead to

$$\frac{\delta \mathcal{L}}{\delta \mathbf{x}(T)} \;=\; \boldsymbol{\nu}(T) \;:=\; 0 \tag{S28}$$

$$\frac{\delta \mathcal{L}}{\delta \mathbf{x_f}(0)} \;=\; 2\lambda \mathbf{x_f}(0) - \boldsymbol{\nu_f}(0) \;:=\; 0 \, , \tag{S29}$$

where the vector $\boldsymbol{\nu_f}$ holds 0's at the entries corresponding to the 0-entries of $\mathbf{x_f}$ (see equation (S24) and (S33)).

The partial first variation with respect to x is now

$$\frac{\delta \mathcal{L}}{\delta \mathbf{x}} \;=\; -\int_0^T \left[\frac{\mathrm{d}\boldsymbol{\nu}}{\mathrm{d}t} + \boldsymbol{\nu} \cdot \frac{\partial \mathbf{F}}{\partial \mathbf{x}} - \frac{2\mathbf{x_f}}{T}\right] \mathrm{d}t \, , \tag{S30}$$

which leads to an *adjoint* equation of motion concerning the Lagrange multipliers $\nu(t)$

$$\frac{\mathrm{d}\boldsymbol{\nu}}{\mathrm{d}t} = -\boldsymbol{\nu} \cdot \frac{\partial \mathbf{F}}{\partial \mathbf{x}} + \frac{2\mathbf{x_f}}{T} \, . \tag{S31}$$

Again, in order to finish the optimization problem, we need to derive the explicit adjoint equations of motion for the power grid model. Therefore, state variables of the dual dynamical equation ($\boldsymbol{\nu}(t)$) in accordance with the actual state variables are separated into two types, with u_i being the counterpart of ϕ_i and v_i being the counterpart of ω_i and thus

$$\boldsymbol{\nu} \;=\; (u_1,\, u_2,\, ...,\, u_N,\, v_1,\, v_2,\, ...,\, v_N) \tag{S32}$$

and

$$\boldsymbol{\nu_f} \;=\; (0,\, 0,\, ...,\, 0,\, v_1,\, v_2,\, ...,\, v_N) \, . \tag{S33}$$

By inserting the dynamical equation (S21) (as F) in equation (S31) we receive the adjoint equations of motion. The first step is the determination of the Jacobian matrix of the dynamical system. The Jacobian

can be outlined as

$$\frac{\partial \mathbf{F}}{\partial \mathbf{x}} = \left(\begin{array}{ccc|ccc} \frac{\partial f_1}{\partial \phi_1} & \cdots & \frac{\partial f_1}{\partial \phi_N} & \frac{\partial f_1}{\partial \omega_1} & \cdots & \frac{\partial f_1}{\partial \omega_N} \\ \vdots & \ddots & \vdots & \vdots & \ddots & \vdots \\ \frac{\partial f_N}{\partial \phi_1} & \cdots & \frac{\partial f_N}{\partial \phi_N} & \frac{\partial f_N}{\partial \omega_1} & \cdots & \frac{\partial f_N}{\partial \omega_N} \\ \frac{\partial g_1}{\partial \phi_1} & \cdots & \frac{\partial g_1}{\partial \phi_N} & \frac{\partial g_1}{\partial \omega_1} & \cdots & \frac{\partial g_1}{\partial \omega_N} \\ \vdots & \ddots & \vdots & \vdots & \ddots & \vdots \\ \frac{\partial g_N}{\partial \phi_1} & \cdots & \frac{\partial g_N}{\partial \phi_N} & \frac{\partial g_N}{\partial \omega_1} & \cdots & \frac{\partial g_N}{\partial \omega_N} \end{array}\right) . \tag{S34}$$

In the illustration, the Jacobian has been divided into two sections. This partitioning refers to the derivation of two different types of evolution equations. The first section is required for the derivation of the dynamical equation for u_i and the second section for v_i.

Dynamical equation for u_i

Each column within the first section of the Jacobian can be completely characterized by four generic partial derivatives. The first two constitute the upper half of an arbitrary column which only contains 0's

$$\frac{\partial f_i}{\partial \phi_i} = 0 \, ; \quad \frac{\partial f_j}{\partial \phi_i} = 0 \, . \tag{S35}$$

The third partial derivative corresponds to a single entry in the lower half of the respective column

$$\frac{\partial g_i}{\partial \phi_i} = -\sum_{j=1}^{N} K_{ji} \cos(\phi_j - \phi_i) \, , \tag{S36}$$

and all remaining entries in the lower half of the particular column are characterized by

$$\frac{\partial g_j}{\partial \phi_i} = K_{ij} \cos(\phi_i - \phi_j) \, . \tag{S37}$$

Now, in order to receive the evolution equation for an arbitrary u_i, the negative adjoint state vector $-\boldsymbol{\nu}$ is multiplied with the respective column of the Jacobian. The dynamical equation for a generic u_i is then

$$\frac{\mathrm{d}u_i}{\mathrm{d}t} = v_i \sum_{j=1}^{N} K_{ji} \cos(\phi_j - \phi_i) - \sum_{j=1}^{N} v_j K_{ij} \cos(\phi_i - \phi_j) \, .$$

Taking into account that the adjacency matrix of the network is symmetric $K_{ij} = K_{ji}$, this expression can be reduced to

$$\frac{\mathrm{d}u_i}{\mathrm{d}t} = \sum_{j=1}^{N} \left[K_{ij} \, (v_i - v_j) \cos(\phi_j - \phi_i) \right] \, . \tag{S38}$$

It should be noted that no additional term is added since the entries in $\mathbf{x_f}$ referring to phases ϕ_i are 0.

Dynamical equation for v_i

The upper half of an exemplary column within the second section of the Jacobian is characterized by

$$\frac{\partial f_i}{\partial \omega_i} = 1 \, ; \quad \frac{\partial f_j}{\partial \omega_i} = 0 \, , \tag{S39}$$

which means that it only includes one single non-zero entry. The same holds true for the lower half of an exemplary column which is defined by

$$\frac{\partial g_i}{\partial \omega_i} = -\alpha \, ; \quad \frac{\partial g_j}{\partial \omega_i} = 0 \tag{S40}$$

By multiplication of the negative dual state vector $-\boldsymbol{\nu}$ with the second section of the Jacobian and adding the additional term $2\omega_i/T$, the dynamical equations for all v_i are received. Accordingly, the general form for the evolution of v_i reads

$$\frac{\mathrm{d}v_i}{\mathrm{d}t} = -u_i + \alpha v_i + \frac{2\omega_i}{T} \, . \tag{S41}$$

Complete adjoint equations of motion

The aggregation of equations (S38) and (S41) now provides the model-specific dual dynamical equation

$$\begin{aligned}
\frac{\mathrm{d}u_i}{\mathrm{d}t} &= \sum_{j=1}^{N} \left[K_{ij} \left(v_i - v_j \right) \cos(\phi_j - \phi_i) \right] \, , \\
\frac{\mathrm{d}v_i}{\mathrm{d}t} &= \alpha v_i \; - \; u_i \; + \; \frac{2\omega_i}{T} \, .
\end{aligned} \tag{S42}$$

The set of equations (S4), (S25), (S28), (S29) and (S42) supplies a complete maximization problem. For a defined value of d, the solution of all equations thereby supplies a maximum, which can only be attained by an iterative method which is described in detail in the following Supplementary Note.

Supplementary Note Iterative Solution of the Optimization Problem

With the introduction of the adjoint dynamical equations and its corresponding state variables, the formulation of the optimization problems for the plant-pollinator network and the power grid model are complete. In order to solve them, an iterative approach is used, which will be presented in detail here. For this purpose, the framework of the iterative method will be outlined first and then details concerning the crucial step of the procedure will be explained. In the following, we will stick to the nomenclature of the power grid model and thus use $\mathbf{x_f}$ and $\boldsymbol{\nu}_\mathbf{f}$, where necessary. By replacing $\mathbf{x_f}$ by $\mathbf{x}$ and $\boldsymbol{\nu}_\mathbf{f}$ by $\boldsymbol{\nu}$, one receives the correct formulation for the plant-pollinator system.

The optimization process for a fixed radius d (fixed perturbation magnitude) can be outlined as follows (adapted from [2]):

Step (I): Initialization

An initial state vector $\mathbf{x}^0(0)$ is chosen in accordance with the initial distance constraint (equation (S6) or equation (S25)). The choice of the direction of $\mathbf{x}^0(0)$ depends on the current stage of the overall search algorithm (see Supplementary Fig. S1 and Methods in the main manuscript).

The calculation of the next iterate $\mathbf{x}^{n+1}(0)$ from the former initial state $\mathbf{x}^n(0)$ is then performed in three steps:

Step (A): Forward integration

Starting with $\mathbf{x}^n(0)$, the equation of motion (equation (S4) or (S21)) is time-integrated forward from $t = 0$ to $t = T$ to receive $\mathbf{x}^n(T)$ and the objective function o^n corresponding to this initial condition (equation (S7) or (S26)).

Step (B): Backward integration

The adjoint equation of motion (equations (S19) and (S20) or equation (S42)) is integrated backwards in time from $t = T$ to $t = 0$ with the initial condition $\boldsymbol{\nu}^n(T) = 2\mathbf{x}^n(T)$ (equation (S9)) or $\boldsymbol{\nu}^n(T) = 0$ (equation (S28)) to receive ${\boldsymbol{\nu}_\mathbf{f}}^n(0)$.

Step (C): Adaptation of initial state

At this point, the only condition of the optimization problem which remains unsatisfied is the variation of the Lagrangian $\mathcal{L}$ with respect to $\mathbf{x_f}(0)$ (equation (S10) or equation (S29)). Accordingly, this equation is applied to adapt the initial state vector. The new guess for the initial state is obtained by simply moving $\mathbf{x}^n(0)$ in the direction of the maximum gradient of $\mathcal{L}$

$$\begin{aligned}\mathbf{x}^{n+1}(0) &= \mathbf{x}^n(0) + \epsilon \left[\frac{\delta\mathcal{L}}{\delta\mathbf{x_f}(0)}\right]^n \\ &= \mathbf{x}^n(0) + \epsilon\,(\,\lambda^*{\mathbf{x_f}}^n(0) - {\boldsymbol{\nu}_\mathbf{f}}^n(0)\,)\;, \qquad \text{(S43)}\end{aligned}$$

where the Lagrange multiplier $\lambda^* = 2\lambda$ is determined in such a way to ensure that the initial distance constraint still holds (equation (S6) or (S25)) for the new initial state and the parameter ϵ can be adjusted to control the size of the adaptation step.

The series of steps (A), (B) and (C) is iterated until a specified convergence criterion indicates the arrival at a maximum of $\mathcal{L}$. In the implemented algorithm, the iteration is stopped as soon as the enhancement of the current objective function is smaller than a predefined small value $o^{n+1}/o^n < 1 + \delta_{crit}$.

(C) Adaptation of initial state

The adaptation of the initial state is the most critical aspect in determining whether and, if so, how fast the optimization process converges to a maximum. In this context, the control of the step size is of particular importance: If adaptation steps are too small, the convergence towards a maximum might take an excessive period of time. On the other hand, if the steps are oversized, the optimization might not converge at all, as the actual maximum is repeatedly overshot. For this reason, the iterative adaptation of $\mathbf{x}^n(0)$ is discussed in more detail, with special emphasis on the step size control.

It has been shown that each new initial state $\mathbf{x}^{n+1}(0)$ is constructed from the current initial state $\mathbf{x}^n(0)$ and the related gradient of the Lagrangian (equation (S43)). However, in order to maintain a valid initial state, the initial distance constraint has to be fulfilled and therefore, the following condition must apply

$$\begin{aligned} d &= ||\, \mathbf{x_f}^{n+1}(0)\, || \\ &= ||\, (1+\epsilon\,\lambda^*)\, \mathbf{x_f}^n(0) - \epsilon\, \boldsymbol{\nu}_\mathbf{f}^n(0)\, ||\, . \end{aligned} \quad \text{(S44)}$$

This can be achieved by determining the Lagrange multiplier λ^* in an appropriate way (taking into account that $||\mathbf{x_f}^n(0)|| = d$)

$$\lambda^*_{1,2} = \frac{\boldsymbol{\nu}_\mathbf{f}^n(0)\cdot\mathbf{x_f}^n(0)}{d^2} - \frac{1}{\epsilon} \pm \sqrt{\frac{1}{\epsilon^2} + \frac{[\,\boldsymbol{\nu}_\mathbf{f}^n(0)\cdot\mathbf{x_f}^n(0)\,]^2}{d^4} - \frac{||\boldsymbol{\nu}_\mathbf{f}^n(0)||^2}{d^2}}\, , \quad \text{(S45)}$$

whereby only the term with positive sign in front of the square root is considered, in order to ensure a positive sign for λ^*. The dependence of λ^* on the step size control parameter ϵ is noteworthy. Since the parameter ϵ appears in the square root of equation (S45), it cannot be chosen arbitrarily. Instead, an upper limit ϵ_{max} is derived which ensures that λ^* is not a complex number (the term in the square root is positive for $\epsilon < \epsilon_{max}$)

$$\epsilon_{max} = \sqrt{\frac{d^4}{d^2\, ||\boldsymbol{\nu}_\mathbf{f}^n(0)||^2 - (\,\boldsymbol{\nu}_\mathbf{f}^n(0)\cdot\mathbf{x_f}^n(0)\,)^2}}\, . \quad \text{(S46)}$$

Consequently, the parameter ϵ should only be varied within the interval $(0, \epsilon_{max}]$ and therefore, a new parameter ϵ^* is introduced which determines the size of the 'original' control parameter ϵ

$$\epsilon = p_c \cdot \epsilon_{max} \cdot \epsilon^*\, , \quad \text{(S47)}$$

in which $p_c \leq 1$ is an arbitrary constant and the new control parameter ϵ^* is limited to the interval $[\epsilon^*_{min}, 1]$.

As the direction for the adaptation is already defined due to the constraint optimization (see equation (S43)), the choice of an optimal step size ϵ^* can be solved by a one-dimensional optimization problem in which the objective function o^{n+1} at the new initial state $\mathbf{x}^{n+1}(0)$ is maximized. First, we determine an interval $[\epsilon^*_a, \epsilon^*_c]$ which is enclosed by two step sizes and which fulfills the following conditions: For three proposed step sizes it holds that $0 \leq \epsilon^*_a < \epsilon^*_b < \epsilon^*_c \leq 1$ and $o_a \geq o^n$, $o_b > o_a$, $o_b > o_c$, where o_a, o_b and o_c are the objective functions corresponding to the initial conditions (equation (S43)) with ϵ^*_a, ϵ^*_b and ϵ^*_c. These conditions assure that the interval includes a local maximum of the objective function. For a graphical representation of the determination of the interval see Supplementary Fig. S2. After having defined this interval, we proceed by alternatingly bisecting the intervals $[\epsilon^*_a, \epsilon^*_b]$ and $[\epsilon^*_b, \epsilon^*_c]$ and choosing the new values of ϵ^*_a, ϵ^*_b and ϵ^*_c in a way that the conditions $\epsilon^*_a < \epsilon^*_b < \epsilon^*_c$, $o_b > o_a$ and $o_b > o_c$ are fulfilled during the whole procedure. The bisection is stopped as soon as the size of the interval $[\epsilon^*_a, \epsilon^*_c]$ falls below a critical limit ϵ^*_{prec}.

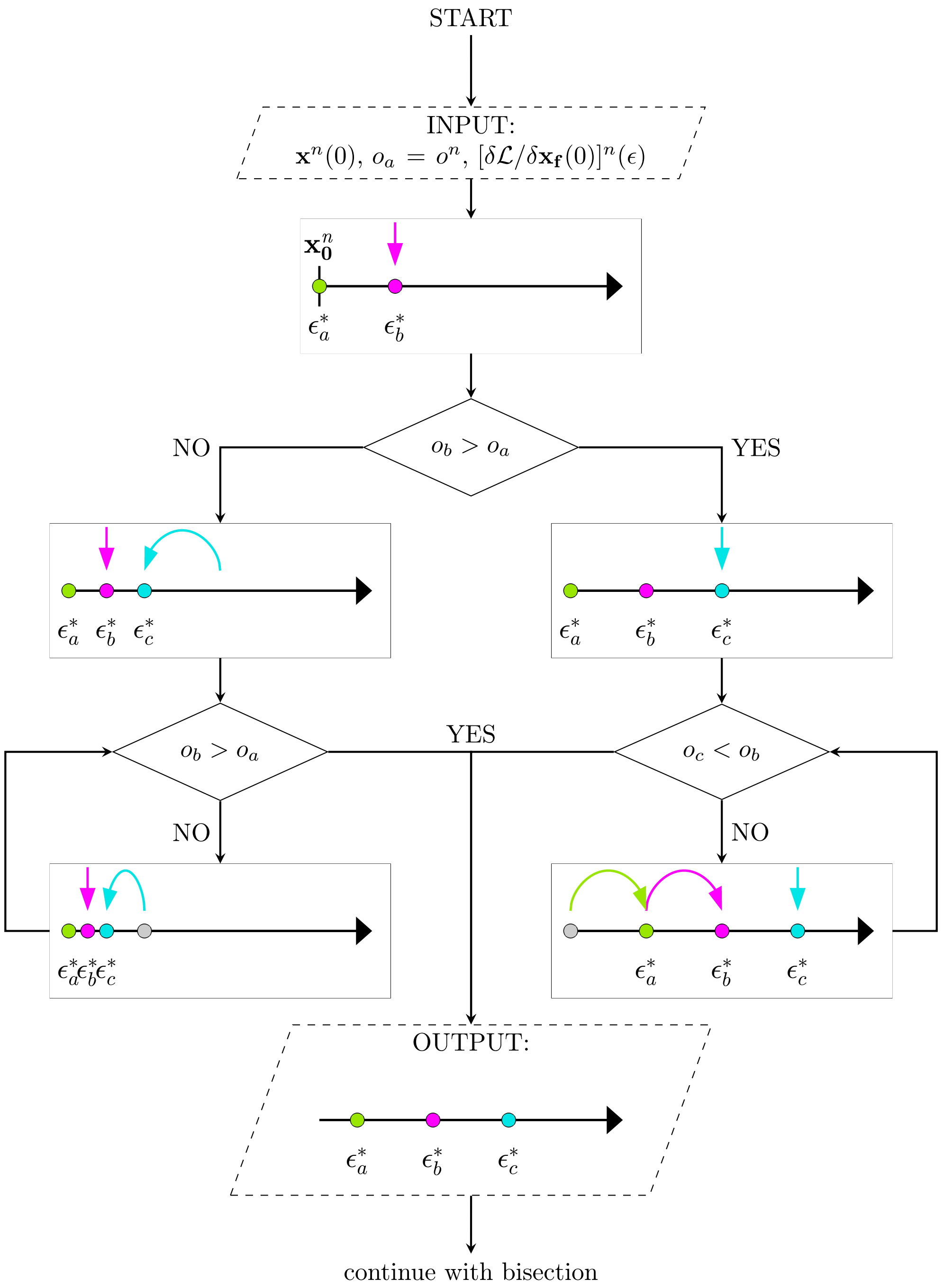

Supplementary Figure S2: **Representation of the determination of the interval for the step size control.** We start by checking whether a certain step size leads to a larger value o_b of the objective function in comparison to the value of the objective function at the current initial condition $\mathbf{x}^n(0)$. *Left branch:* If it does not, we need to reduce the step size and thus the location of ϵ_b^* is shifted towards $\mathbf{x}^n(0)$ and the former location of ϵ_b^* now depicts the right edge of the interval which is ϵ_c^*. We then continue by shifting the interval to the left until $o_b > o_a$ or the distance between ϵ_c^* and ϵ_a^* falls below ϵ_{crit}^*. *Right branch:* If the initial ϵ_b^* provides a value o_b which is larger than the initial value of the objective function, we continue by placing ϵ_c^* on the right side of ϵ_b^*. Now, if the value o_c corresponding to ϵ_c^* is smaller than o_b, we have an interval which contains a local maximum. If not, we continue by shifting the interval to the right until $o_c < o_b$. *Bottom:* Finally, we end up with an interval in which we can locate the local maximum by alternatingly bisecting both subintervals $[\epsilon_a^*, \epsilon_b^*]$ and $[\epsilon_b^*, \epsilon_c^*]$. This figure was generated using the TikZ package from LaTeX and MATLAB version R2017a (`https://www.mathworks.com/`).

parameter	default value in PP Network / Power Grid	meaning
**Stage I: Random Global Initialization**		
d_{max}	$0.999 \cdot P^*$ / 20.0	initial maximum perturbation magnitude; P^* is equal to every entry of $\mathbf{X_0}$
n_{trials}	1* / 50	number of trials; * search starts with any fatal initial shock
**Stage II: Non-Random Local Search**		
Δd	(0.1, 0.05, 0.01, 0.001, 0.05, 0.05, 0.001) / (0.1, 0.05, 0.01, 0.001, 0.001, 0.001, 0.0001)	step sizes for reduction of d; every time a non-fatal shock is reached, Δd is adapted
T_{ini}	5.0 / [5.0, 20.0]*	initial integration time; * T_{ini} is randomly drawn from a uniform distribution
T_+	(0.0, 0.0, 0.0, 0.0, 2.0, 3.0, 0.0) / (0.0, 0.0, 0.0, 0.0, 5.0, 5.0, 5.0)	increase of integration time; every time Δd is adapted, T is increased
n_{in}	1 / (3, 3, 10, 15, 15, 15, 15)*	critical number of local optima located within the basin of $\mathbf{X_0}$; * depends on Δd
δ_{crit}	10^{-6} / 10^{-5}	critical enhancement of o (convergence criterion)
p_c	0.1 / 0.1	arbitrary factor ($p_c \leq 1$) for step size control
ϵ^*_{min}	10^{-5} / 10^{-3}	critical minimum value of ϵ^* (step size control)
ϵ^*_{prec}	10^{-4} / 10^{-3}	precision for determination of ϵ^*
ϵ^*_a, ϵ^*_b, ϵ^*_b	0.0, 0.1, 0.2 / 0.0, 0.1, 0.2	initial values for the determination of ϵ^*

Supplementary Table S1: **Default parameter settings for the search algorithm.** For some of the search runs, the parameters were slightly varied (e.g. higher values for n_{trials}). The depicted parameters represent the default parameter settings which we chose in order to attain different local MiFaS as well.

Supplementary Note The Choice of Integration Times

One of the most delicate choices in the constraint optimization procedure concerns the integration time T. Obviously, the use of short integration times is advantageous as it keeps the computation time short. But even more importantly, short integration times smooth differences between the magnifications caused by adjacent initial perturbations (landscape of objective function, see Supplementary Fig. S3 and Supplementary Fig. S4) and thus allow the application of the optimization even in highly complex landscapes of basins of attractions. This is of special importance in the Great Britain power grid in which the inspection of long integration times exposes a highly complex basin structure. Accordingly, in order to apply the constraint optimization for initial conditions which are not located close to the boundary of the basin of $\mathbf{X_0}$, we need to use short integration times to approach the basin boundary of the basin of $\mathbf{X_0}$ (Supplementary Fig. S4). However, the use of short integration times is crucial in the early phase of the optimization for systems with less complex basin structures as well. For instance, for a bundle of adjacent initial conditions which are far off the basin boundary of $\mathbf{X_0}$, no signifcant difference in the distances to the desired state after a long integration time can be detected as all of them might already have reached an undesired attractor (see Supplementary Fig. S3d,e).

However, during the optimization procedure (see e.g. Supplementary Fig. S1c,d) the sets of inspected initial conditions get closer to the basin boundary of the basin of $\mathbf{X_0}$. Accordingly, the associated trajectories stay in the vicinity of the basin boundary for longer times and therefore, in order to indicate the perturbations which diverge the fastest, we need to apply longer integration times T. We solve this issue by starting with a relatively short integration time which we increase every time Δd is decreased (see Supplementary Fig. S1c,d and Supplementary Table S1).

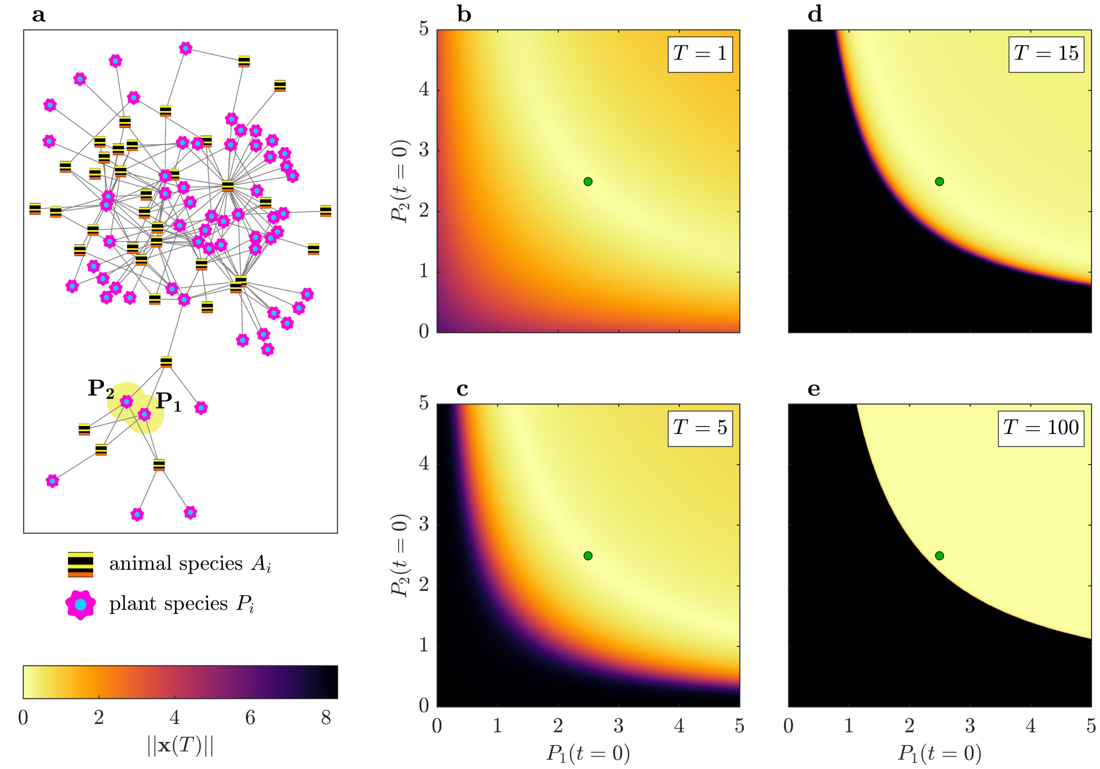

Supplementary Figure S3: **Cross sections of the objective function landscape in dependence on the integration time for an exemplary plant-pollinator network .** (a) In order to create a cross section of the Euclidean distance to the desired state $\mathbf{X_0}$ after integration time T, two plant species are chosen whose initial abundances $P_1(t = 0)$ and $P_2(t = 0)$ are varied. (b-e) Euclidean distances to the desired state after integration time T in dependence on the initial state. The initial state equals the desired state for all species, except for species P_1 and P_2. Placement of the vertices in (a) is based on the Kamada-Kawai algorithm [3] obtained from python-igraph version 0.7.1 (`https://igraph.org/`). This figure was generated using MATLAB version R2017a (`https://www.mathworks.com/`).

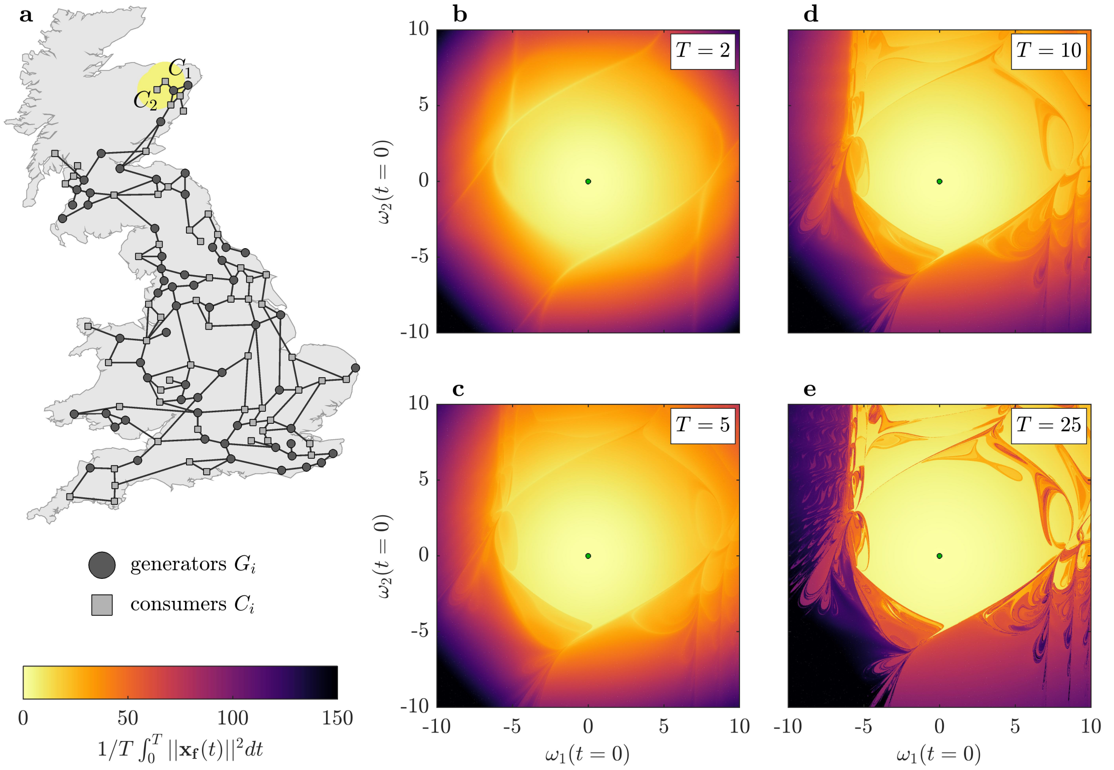

Supplementary Figure S4: **Cross sections of the objective function landscape in dependence on the integration time for the Great Britain power grid.** (a) In order to create a cross section of the mean Euclidean distance to the desired state $\mathbf{X_0}$ over the integration time T, two consumers are chosen $C_1(t = 0)$ and $C_2(t = 0)$ whose initial frequencies ω_1 and ω_2 are varied. (b-e) Mean Euclidean distances to the desired state over the integration time T in dependence on the initial state. The initial state equals the desired state for all oscillators, except for consumers C_1 and C_2. This figure was generated using MATLAB version R2017a (`https://www.mathworks.com/`).

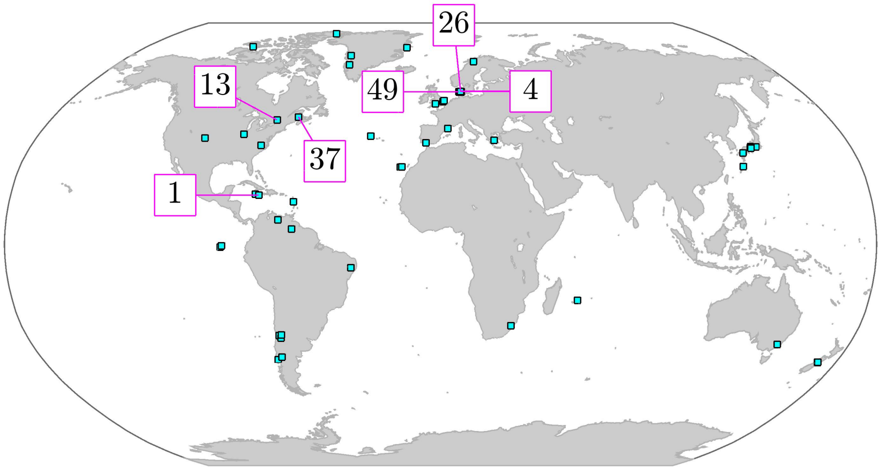

Supplementary Figure S5: **Location of the examined plant-pollinator networks.** The locations of the 59 plant-pollinator networks for which the MiFaS have been calculated are marked by cyan squares. In addition, the location of the six plant-pollinator networks for which the direction of the MiFaS has been depicted in the main text are indicated. The numbering refers to the sorting of the 59 networks according to the absolute value of their MiFaS in ascending order. This figure was generated using the mapping toolbox of MATLAB version R2017a (`https://www.mathworks.com/`).

no.	ID *(web of life)*	location of study	species (original)	species (considered)	reference/ *source*
1	M_PL_035	Morant Point, Jamaica	97	97	[4]
2	M_PL_023	Rio Blanco, Mendoza, Argentina	95	90	[5]
3	M_PL_022	Laguna Diamante, Mendoza, Argentina	66	66	[5]
4	M_PL_037	Hestehaven, Denmark	50	50	[6]
5	M_PL_054	Kyoto City, Japan	431	414	[7]
6	M_PL_039	Tenerife, Canary Islands	68	68	[8]
7	M_PL_044	Amami-Ohsima Island, Japan	719	712	[9]
8	M_PL_053	Mt. Yufu, Japan	393	364	[10]
9	M_PL_057	Kibune, Kyoto, Japan	997	997	[11]
10	M_PL_031	Canaima Nat. Park, Venezuela	97	91	[12]
11	M_PL_056	Mt. Kushigata, Yamanashi Pref., Japan	456	456	[13]
12	M_PL_005	Pikes Peak, Colorado, USA	371	361	[14]
13	M_PL_033	Ottawa, Canada	47	47	[15]
14	M_PL_055	Nakaikemi marsh, Fukui Prefecture, Japan	259	253	[16]
15	M_PL_028	Cass, New Zealand	180	180	[17]
16	M_PL_012	Garajonay, Gomera, Spain	84	84	[18]
17	M_PL_002	Cordón del Cepo, Chile	107	103	[19]
18	M_PL_030	Guarico State, Venezuela	81	70	[20]
19	M_PL_021	Ashu, Kyoto, Japan	768	766	[21]
20	M_PL_027	Arthur's Pass, New Zealand	78	75	[22]
21	M_PL_009	Latnjajaure, Abisko, Sweden	142	142	[23]
22	M_PL_013	KwaZulu-Natal region, South Africa	65	65	[24]
23	M_PL_003	Cordón del Cepo, Chile	61	61	[19]
24	M_PL_020	Hazen Camp, Ellesmere Island, Canada	111	109	[25]
25	M_PL_026	Galapagos	159	150	[26]
26	M_PL_038	Hestehaven, Denmark	50	50	[6]
27	M_PL_051	Nahuel Huapi National Park, Argentina	104	104	[27]
28	M_PL_006	Hickling, Norfolk, UK	78	78	[28]
29	M_PL_040	Windsor, The Cockpit Country, Jamaica	72	70	[29]
30	M_PL_029	Craigieburn, New Zealand	167	167	[30]
31	M_PL_001	Cordón del Cepo, Chile	185	177	[19]
32	M_PL_036	Flores, Açores	22	22	[18]
33	M_PL_019	Snowy Mountains, Australia	125	123	[31]
34	M_PL_045	Uummannaq Island, Greenland	43	41	[32]
35	M_PL_049	Denmark	262	262	[33]
36	M_PL_034	Chiloe, Chile	154	151	[34]
37	M_PL_004	Central New Brunswick, Canada	114	112	[35]
38	M_PL_007	Shelfanger, Norfolk, UK	52	50	[28]
39	M_PL_014	Hazen Camp, Ellesmere Island, Canada	110	108	[36]
40	M_PL_041	Syndicate, Dominica	74	70	[29]
41	M_PL_050	Tenerife, Canary Islands	49	49	[37]
42	M_PL_016	Doñana Nat. Park, Spain	205	205	[38]
43	M_PL_025	North Carolina, USA	57	57	[39]
44	M_PL_043	Hestehaven, Denmark	110	110	[6]
45	M_PL_024	Melville Island, Canada	29	22	[40]
46	M_PL_047	Isenbjerg	205	205	[41]
47	M_PL_018	Hestehaven, Denmark	144	144	[18]
48	M_PL_015	Daphní, Athens, Greece	797	793	[42]
49	M_PL_048	Denmark	266	266	[41]
50	M_PL_032	Brownfield, Illinois, USA	40	40	[43]
51	M_PL_042	Puerto Villamil, Isabela Island, Galapagos	18	16	[44]
52	M_PL_052	Tundra, Greenland	54	52	[45]
53	M_PL_058	Parc Natural del Cap de Creus	113	111	[46]
54	M_PL_008	Tenerife, Canary Islands	49	49	[47]
55	M_PL_010	Zackenberg	107	107	[48]
56	M_PL_011	Mauritius Island	27	27	[49]
57	M_PL_017	Bristol, England	104	104	[50]
58	M_PL_046	Denmark	60	60	[51]
59	M_PL_059	Parque Nacional do Catimbau	26	26	[52]

Supplementary Table S2: **Overview of the dataset of plant-pollinator networks.** From every dataset, the largest connected component is taken as the network topology while all other components are neglected. Accordingly, the examined networks are sometimes slightly smaller than the original network data.

Supplementary Note Computation Time

Supplementary Fig. S6 illustrates that - for plant-pollinator networks - the mean computation time to complete one run of the search algorithm (as outlined in the main manuscript and Supplementary Fig. S1) does not only depend on network size but also on properties of the basin landscape. It must be noted that one run of the search algorithm corresponds to the computation of one local minimal fatal shock and thus the figure does not take into account that for systems with a high number of state variables N more runs are needed to assure a good estimate for the global MiFaS than for systems with fewer state variables.

For networks exhibiting a small or medium MiFaS, the computation time increases linearly with network size (number of species N in the network). However, we find that for networks associated with large MiFaS computation times strongly deviate from this trend and are much longer than expected. These comparatively long computation times are associated to stage I of the search algorithm. In fact, the random global initialization is not adapted to the case of a large basin of attraction of the desired state. If the basin of the desired state spans the majority of the tested phase space, most of the random initial conditions will correspond to non-fatal perturbations and thus many trials are needed to find one fatal initial perturbation. Since testing whether a perturbation is fatal or non-fatal requires long integration times - in comparison to the short integration times within the optimization of stage II - the computation time drastically increases.

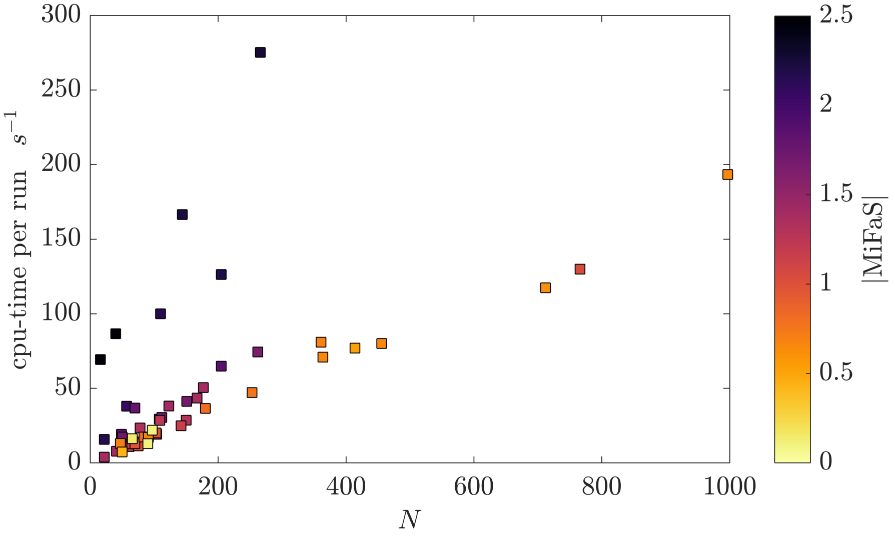

Supplementary Figure S6: **Mean computation time to compute one local MiFaS.** Mean computation time to complete one run of the search algorithm depending on network size and the magnitude of the global MiFaS for 50 plant-pollinator networks. We excluded those networks for which $|\text{MiFaS}| > 2.4$ and the second largest network (M_PL_015) for which $|\text{MiFaS}| = 2.245$. This figure was generated using MATLAB version R2017a (`https://www.mathworks.com/`).

Supplementary Note Resistence Centralities in the Power Grid

As an exemplary centrality index which captures the core-periphery distribution of vertices within an oscillatory network, we choose to present the resistance centrality [53] for the GB power grid. This centrality index has proven efficient in identifying the most peripheral nodes as the ones generating the largest excursions after being disturbed [54]. The following description of the resistance centrality follows the more elaborated descriptions in [53, 54].

The coupling of oscillators within the power grid model (equation (S21)) can be captured with the Laplacian matrix $\mathbf{L}$ with $\mathbf{L}_{ij} = -K_{ij}$ if $i \neq j$ and $\mathbf{L}_{ii} = \sum_k K_{ik}$. The centrality index proposed by [53, 54] is based on the resistance distance [55]. The resistance distance Ω_{ij} is defined as the effective resistence between two nodes within an electrical network. In order to obtain Ω_{ij} for an arbitrary network, one imagines it as an electrical network in which each edge corresponds to a resistor whose capacity is given by the inverse of the weight of this edge. Using the Laplacian of the network, the resistance distance can be expressed as

$$\Omega_{ij} = \mathbf{L}^+_{ii} + \mathbf{L}^+_{jj} - \mathbf{L}^+_{ij} - \mathbf{L}^+_{ji} \tag{S48}$$

where $\mathbf{L}^+$ is the Moore-Penrose pseudoinverse of $\mathbf{L}$.

The resistance centrality index of node i is then obtained as the inverse of the average resistance distance of node i to all other vertices of the network

$$C_1(i) = \left[n^{-1} \sum_{j=1}^{N} \Omega_{ij} \right]^{-1} . \tag{S49}$$

Large values of $C_1(i)$ indicate a central position and small values a peripheral position of the vertex i.

In order to include not only information on the network topology but also on the operational state $\mathbf{x_0}$ (see equation (S23)) of the power grid, one exchanges the Laplacian by the weighted Laplacian $\mathbf{L}^*$ whose entries are

$$\mathbf{L}^*_{ij} = \begin{cases} -K_{ij} \cos(\phi^*_i - \phi^*_j), & i \neq j \, , \\ \sum_k K_{ik} \cos(\phi^*_i - \phi^*_k), & i = j \, . \end{cases} \tag{S50}$$

This weighted matrix $\mathbf{L}^*$ differs only significantly from the unweighted Laplacian $\mathbf{L}$ if phase differences between connected nodes are large (corresponding to high loads on the respective transmission line). By using $\mathbf{L}^*$ instead of $\mathbf{L}$, one obtains another resistance centrality C^*_1. The distribution of centrality indices C^*_1 for the nodes of the GB power grid is illustrated in Supplementary Fig. S7.

Tyloo et al. [53] found that a system's response to different types of perturbation depends on the overlap between the particular perturbation and the eigenmodes of the weighted Laplacian matrix. In reference to their work, we checked whether the identified MiFaS matches the direction of one of the eigenmodes by calculating the scalar product of the direction of the MiFaS and all eigenmodes of $\mathbf{L}^*$ (Supplementary Fig. S8). Noteworthy, we find that the MiFaS is orthogonal to the first eigenmode $u_1 = (1, 1, ..., 1)/\sqrt{N}$ with eigenvalue $\lambda_1 = 0$ (eigenmodes are sorted from 1 to N in ascending order of their eigenvalues) and thus orthogonal to a neutral perturbation which would affect all oscillators in the same way. Apart from that, the MiFaS shows some degree of alignment with some of the eigenmodes (especially eigenmode 3 and 4) but does not perfectly match any of them.

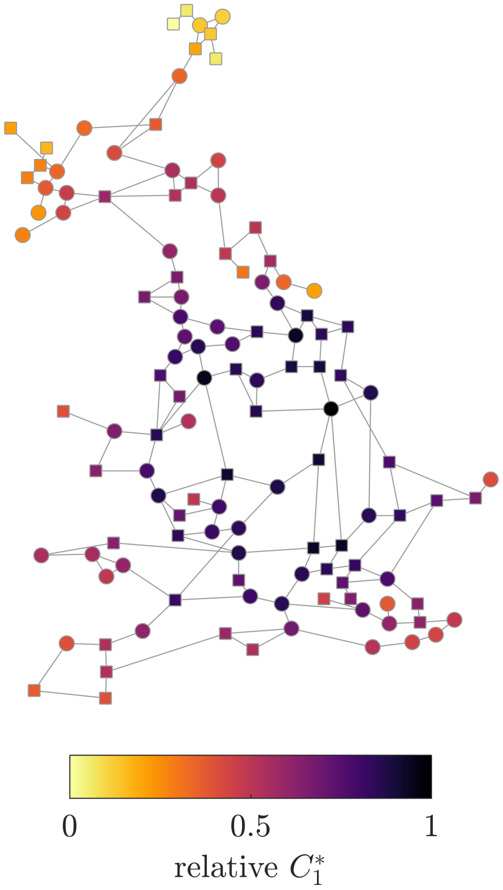

Supplementary Figure S7: **Relative resistence centrality within the Great Britain power grid.** The resistence centrality C_1^* based on the weighted Laplacian $\mathbf{L}^*$ is scaled to the interval [0, 1]. Squares portray consumers and circles generators. This figure was generated using MATLAB version R2017a (`https://www.mathworks.com/`).

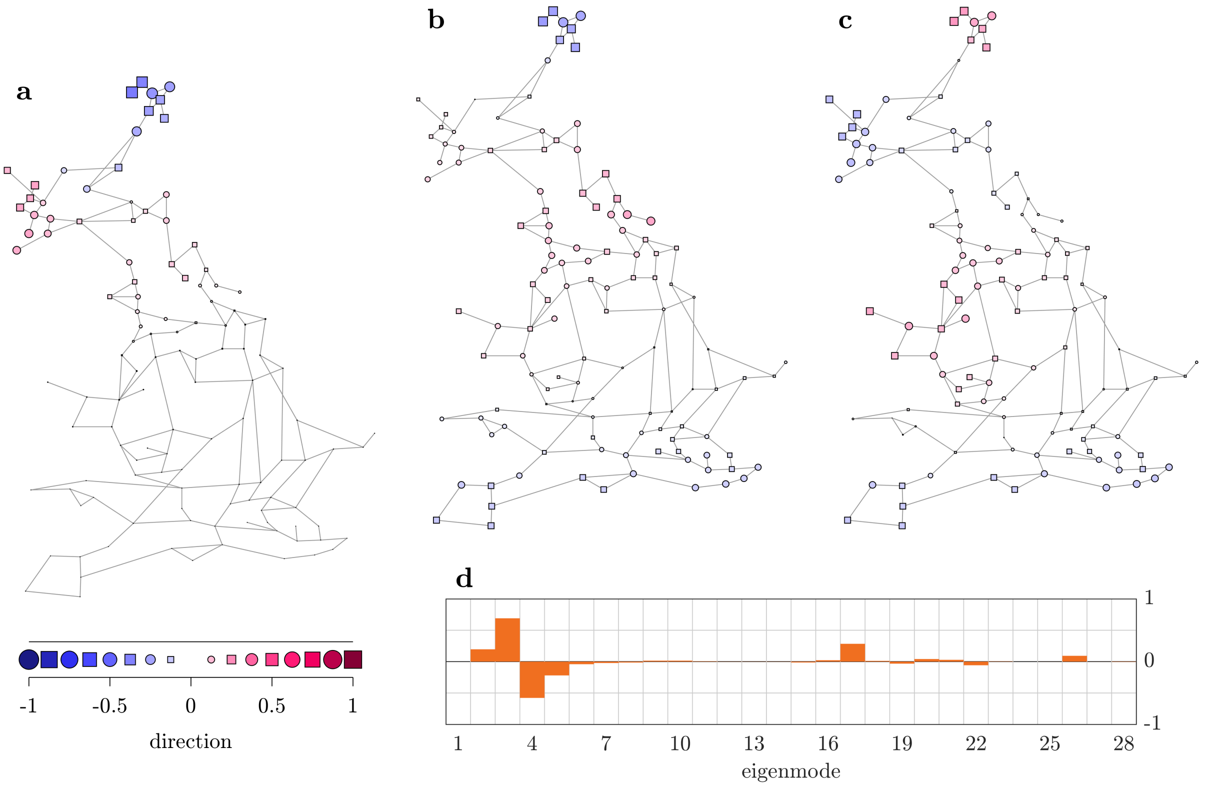

Supplementary Figure S8: **Relation between the MiFaS and the eigenmodes of the weighted Laplacian.** (a) Direction of the MiFaS. The perturbation vector is scaled to a length of 1. The contribution of each element of the vector (node in the network) to the overall perturbation is represented by the area and the color saturation of the respective squares and circles. Squares portray consumers and circles generators. (b) Third eigenmode of the weighted Laplacian $\mathbf{L}^*$. (c) Fourth eigenmode of the weighted Laplacian. (d) Scalar products of the direction vector of the MiFaS and each of the first 28 eigenmodes of the weighted Laplacian. This figure was generated using MATLAB version R2017a (`https://www.mathworks.com/`).

Supplementary References

[1] Filatrella, G., Nielsen, A. H. & Pedersen, N. F. Analysis of a power grid using a Kuramoto-like model. *The European Physical Journal B-Condensed Matter and Complex Systems* **61**, 485–491 (2008).

[2] Kerswell, R., Pringle, C. & Willis, A. An optimization approach for analysing nonlinear stability with transition to turbulence in fluids as an exemplar. *Reports on Progress in Physics* **77**, 085901 (2014).

[3] Kamada, T., Kawai, S. *et al.* An algorithm for drawing general undirected graphs. *Information processing letters* **31**, 7–15 (1989).

[4] Percival, M. Floral ecology of coastal scrub in southeast jamaica. *Biotropica* 104–129 (1974).

[5] Medan, D. *et al.* Plant-pollinator relationships at two altitudes in the andes of mendoza, argentina. *Arctic, Antarctic, and Alpine Research* **34**, 233–241 (2002).

[6] Montero, A. *The ecology of three pollination networks.* Master's thesis, University of Aarhus, Denmark (2005).

[7] Kakutani, T., Inoue, T., Kato, M. & Ichihashi, H. Insect-flower relationship in the campus of kyoto university, kyoto: an overview of the flowering phenology and the seasonal pattern of insect visits. (1990).

[8] Stald, L., Valido, A. & Olesen, J. *Struktur og dynamik i rum og tid at et bestøvningsnetværk på Tenerife, De Kanariske Øer.* Master's thesis, University of Aarhus, Denmark (2003).

[9] Kato, M. Anthophilous insect community and plant-pollinator interactions on amami islands in the ryukyu archipelago, japan (original paper). (2000).

[10] Yamazaki, K. & Kato, M. Flowering phenology and anthophilous insect community in a grassland ecosystem at mt. yufu, western japan. (2003).

[11] Inoue, T., Kato, M., Kakutani, T., Suka, T. & Itino, T. Insect-flower relationship in the temperate deciduous forest of kibune, kyoto: an overview of the flowering phenology and the seasonal pattern of insect visits. (1990).

[12] Ramirez, N. Biología de polinización en una comunidad arbustiva tropical de la alta guayana venezolana. *Biotropica* 319–330 (1989).

[13] Kato, M., Matsumoto, M. & Kato, T. Flowering phenology and anthophilous insect community in the cool-temperate subalpine forests and meadows at mt. kushigata in the central part of japan. (1993).

[14] Clements, F. E. & Long, F. L. Experimental pollination; an outline of the ecology of flowers and insects 5. pollinators and flowers visited & 6. flowers and their visitors. (1923).

[15] Small, E. Insect pollinators of the mer bleue peat bog of ottawa. *Canadian field-naturalist* (1976).

[16] Kato, M. & Miura, R. Flowering phenology and anthophilous insect community at a threatened natural lowland marsh at nakaikemi in tsuruga, japan. (1996).

[17] Primack, R. B. Insect pollination in the new zealand mountain flora. *New Zealand Journal of Botany* **21**, 317–333 (1983).

[18] Olesen, J. M. *unpublished* .

[19] Arroyo, M. T. K., Primack, R. & Armesto, J. Community studies in pollination ecology in the high temperate andes of central chile. i. pollination mechanisms and altitudinal variation. *American journal of botany* **69**, 82–97 (1982).

[20] Ramirez, N. & Brito, Y. Pollination biology in a palm swamp community in the venezuelan central plains. *Botanical Journal of the Linnean Society* **110**, 277–302 (1992).

[21] Kato, M., Kakutani, T., Inoue, T. & Itino, T. Insect-flower relationship in the primary beech forest of ashu, kyoto: an overview of the flowering phenology and the seasonal pattern of insect visits. (1990).

[22] Primack, R. B. Insect pollination in the new zealand mountain flora. *New Zealand Journal of Botany* **21**, 317–333 (1983).

[23] Elberling, H. & Olesen, J. M. The structure of a high latitude plant-flower visitor system: the dominance of flies. *Ecography* **22**, 314–323 (1999).

[24] Ollerton, J., Johnson, S. D., Cranmer, L. & Kellie, S. The pollination ecology of an assemblage of grassland asclepiads in south africa. *Annals of Botany* **92**, 807–834 (2003).

[25] Kevan, P. G. High arctic insect-flower relations the interrelationships of arthropods and flowers at lake hazen, ellesmere island, nwt, canada. (2002).

[26] McMullen, C. Flower-visiting insects of the galapagos islands. *The Pan-Pacific entomologist (USA)* (1993).

[27] Vázquez, D. P. Interactions among introduced ungulates, plants, and pollinators: a field study in the temperate forest of the southern andes. (2002).

[28] Dicks, L., Corbet, S. & Pywell, R. Compartmentalization in plant–insect flower visitor webs. *Journal of Animal Ecology* **71**, 32–43 (2002).

[29] Ingversen, T. *Plant-pollinator interactions on Jamaica and Dominica: the centrality, asymmetry and modularity of networks*. Master's thesis, University of Aarhus, Denmark (2006).

[30] Primack, R. B. Insect pollination in the new zealand mountain flora. *New Zealand Journal of Botany* **21**, 317–333 (1983).

[31] Inouye, D. W. & Pyke, G. H. Pollination biology in the snowy mountains of australia: comparisons with montane colorado, usa. *Australian Journal of Ecology* **13**, 191–205 (1988).

[32] Lundgren, R. & Olesen, J. M. The dense and highly connected world of greenland's plants and their pollinators. *Arctic, Antarctic, and Alpine Research* **37**, 514–520 (2005).

[33] Bek, S. *A pollination network from a Danish forest meadow*. Master's thesis, University of Aarhus, Denmark (2006).

[34] Smith-Ramírez, C., Martinez, P., Nunez, M., González, C. & Armesto, J. J. Diversity, flower visitation frequency and generalism of pollinators in temperate rain forests of chiloé island, chile. *Botanical Journal of the Linnean Society* **147**, 399–416 (2005).

[35] Barrett, S. C. & Helenurm, K. The reproductive biology of boreal forest herbs. i. breeding systems and pollination. *Canadian Journal of Botany* **65**, 2036–2046 (1987).

[36] Hocking, B. Insect-flower associations in the high arctic with special reference to nectar. *Oikos* 359–387 (1968).

[37] Stald, L., Valido, A. & Olesen, J. *Struktur og dynamik i rum og tid at et bestøvningsnetværk på Tenerife, De Kanariske Øer*. Master's thesis, University of Aarhus, Denmark (2003).

[38] Herrera, J. Pollination relationships in southern spanish mediterranean shrublands. *The Journal of Ecology* 274–287 (1988).

[39] Motten, A. Pollination ecology of the spring wildflower community in the deciduous forests of piedmont north carolina (hepatica, erythronium, claytonia). (1984).

[40] Mosquin, T. Observations on the pollination biology of plants on melville island, nwt. *Can. Fld Nat.* **81**, 201–205 (1967).

[41] Dupont, Y. L. & Olesen, J. M. Ecological modules and roles of species in heathland plant–insect flower visitor networks. *Journal of Animal Ecology* **78**, 346–353 (2009).

[42] Petanidou, T. *Pollination ecology in a phryganic ecosystem*. Bachelor's thesis, University of Thessaloniki (1991).

[43] Schemske, D. W. *et al.* Flowering ecology of some spring woodland herbs. *Ecology* **59**, 351–366 (1978).

[44] Philipp, M., Böcher, J., R. Siegismund, H. & R. Nielsen, L. Structure of a plant-pollinator network on a pahoehoe lava desert of the galápagos islands. *Ecography* **29**, 531–540 (2006).

[45] Witt, P. Bachelor's thesis, University of Aarhus, Denmark (1998).

[46] Bartomeus, I., Vilà, M. & Santamaría, L. Contrasting effects of invasive plants in plant–pollinator networks. *Oecologia* **155**, 761–770 (2008).

[47] Dupont, Y. L., Hansen, D. M. & Olesen, J. M. Structure of a plant–flower-visitor network in the high-altitude sub-alpine desert of tenerife, canary islands. *Ecography* **26**, 301–310 (2003).

[48] Elberling, H. & Olesen, J. M. *unpublished* .

[49] Olesen, J. M., Eskildsen, L. I. & Venkatasamy, S. Invasion of pollination networks on oceanic islands: importance of invader complexes and endemic super generalists. *Diversity and Distributions* **8**, 181–192 (2002).

[50] Memmott, J. The structure of a plant-pollinator food web. *Ecology letters* **2**, 276–280 (1999).

[51] Bundgaard, M. *Tidslig og rumlig variation i et plante-bestøvernetværk*. Master's thesis, University of Aarhus, Denmark (2003).

[52] Bezerra, E. L., Machado, I. C. & Mello, M. A. Pollination networks of oil-flowers: a tiny world within the smallest of all worlds. *Journal of Animal Ecology* **78**, 1096–1101 (2009).

[53] Tyloo, M. & Jacquod, P. Global robustness versus local vulnerabilities in complex synchronous networks. *Physical Review E* **100**, 032303 (2019).

[54] Tyloo, M., Pagnier, L. & Jacquod, P. The key player problem in complex oscillator networks and electric power grids: Resistance centralities identify local vulnerabilities. *Science Advances* **5**, eaaw8359 (2019).

[55] Klein, D. J. & Randić, M. Resistance distance. *Journal of mathematical chemistry* **12**, 81–95 (1993).
